# Signature motifs of GDP polyribonucleotidyltransferase, a non-segmented negative strand RNA viral mRNA capping enzyme, domain in the L protein are required for covalent enzyme–pRNA intermediate formation

**DOI:** 10.1093/nar/gkv1286

**Published:** 2015-11-23

**Authors:** Julie Neubauer, Minako Ogino, Todd J. Green, Tomoaki Ogino

**Affiliations:** 1 Department of Molecular Biology and Microbiology, Case Western Reserve University School of Medicine, Cleveland, OH 44106, USA; 2 Department of Microbiology, School of Medicine, University of Alabama at Birmingham, Birmingham, AL 35294, USA

## Abstract

The unconventional mRNA capping enzyme (GDP polyribonucleotidyltransferase, PRNTase; block V) domain in RNA polymerase L proteins of non-segmented negative strand (NNS) RNA viruses (e.g. rabies, measles, Ebola) contains five collinear sequence elements, Rx(3)Wx(3–8)ΦxGxζx(P/A) (motif A; Φ, hydrophobic; ζ, hydrophilic), (Y/W)ΦGSxT (motif B), W (motif C), HR (motif D) and ζxxΦx(F/Y)QxxΦ (motif E). We performed site-directed mutagenesis of the L protein of vesicular stomatitis virus (VSV, a prototypic NNS RNA virus) to examine participation of these motifs in mRNA capping. Similar to the catalytic residues in motif D, G1100 in motif A, T1157 in motif B, W1188 in motif C, and F1269 and Q1270 in motif E were found to be essential or important for the PRNTase activity in the step of the covalent L-pRNA intermediate formation, but not for the GTPase activity that generates GDP (pRNA acceptor). Cap defective mutations in these residues induced termination of mRNA synthesis at position +40 followed by aberrant stop–start transcription, and abolished virus gene expression in host cells. These results suggest that the conserved motifs constitute the active site of the PRNTase domain and the L-pRNA intermediate formation followed by the cap formation is essential for successful synthesis of full-length mRNAs.

## INTRODUCTION

The 5′-terminal cap structure (cap 0, m^7^G[5′]ppp[5′]N-) of eukaryotic mRNAs is composed of *N*^7^-methylguanosine (m^7^G) linked to the 5′-end of mRNA through the 5′-5′ triphosphate (ppp) bridge, and is essential for various steps of mRNA metabolism including stability and translation [Reviewed in (1–4)]. The cap 0 structure is formed on pre-mRNAs by nuclear mRNA capping enzyme with the RNA 5′-triphosphatase (RTPase) and mRNA guanylyltransferase (GTase) activities followed by mRNA (guanine-*N*^7^)-methyltransferase (MTase). In higher eukaryotes, the cap 0 structure is further methylated at the ribose-2′-*O* position of the first nucleoside of mRNA by mRNA (nucleoside-2′-*O*)-MTase to generate the cap 1 structure (m^7^G[5′]ppp[5′]Nm). Recently, 2′-*O*-methylation of the cap structure was found to be required for avoiding anti-viral innate immunity through the IFIT-1, Mda5 and RIG-I pathways (5–9). Thus, many eukaryotic viruses need to possess the fully methylated cap 1 structure on their mRNAs to efficiently produce viral proteins in infected cells. However, the molecular mechanisms of the cap formation by some RNA viral enzymes are significantly different from those by eukaryotic nuclear enzymes (10–12).

Using our newly developed *in vitro* RNA capping assay with an oligo-RNA as a substrate, we have shown that the multifunctional large (L) protein (2109 amino acids) of vesicular stomatitis virus (VSV), a prototypic non-segmented negative strand (NNS) RNA virus belonging to the *Vesiculovirus* genus of the *Rhabdoviridae* family in the order *Mononegavirales*, catalyzes unconventional mRNA capping reactions with a novel set of enzymatic activities [Reviewed in (13,14)]. First, the L protein-associated guanosine 5′-triphosphatase (GTPase) activity removes the γ-phosphate of GTP to produce GDP, which in turn serves as an acceptor for 5′-monophosphorylated RNA (pRNA) (11,15). Then, a GDP polyribonucleotidyltransferase (PRNTase; systematic name, 5′-triphospho-mRNA:GDP 5′-phosphopolyribonucleotidyltransferase; EC 2.7.7.88) domain in the L protein specifically reacts with a viral mRNA start-sequence (5′-AACAG) with a 5′-triphosphate group (pppRNA, pRNA donor) to form a covalent complex with the 5′-monophosphate end of the RNA (called L-pRNA intermediate) most probably with concomitant release of inorganic pyrophosphate (PP_i_) (11,16).

We have demonstrated that pRNA is covalently linked to the *N*^ϵ2^ position of a histidine residue at position 1227 (H1227) in the histidine-arginine (HR) motif of the VSV L protein with a phosphoamide bond and is subsequently transferred to GDP (pRNA acceptor) to form GpppRNA (16). It has been also shown that pRNA linked to H1227 is transferred to PP_i_ to regenerate pppRNA, indicating that the covalent intermediate formation is a reversible reaction (16). Furthermore, both the residues of the HR motif were found to be critical for the intermediate formation with the VSV and Chandipura virus (CHPV, *Vesiculovirus*) L proteins (16,17). Alignment of amino acid sequences of ∼90 NNS RNA viral L proteins revealed that the HR motif is strikingly conserved in L proteins of NNS viruses belonging to the different families, *Rhabdoviridae* (e.g. rabies), *Paramyxoviridae* (e.g. measles), *Filoviridae* (e.g. Ebola) and *Bornaviridae* (e.g. Borna disease), except for fish novirhabdoviruses (16). Recently, we have found that the HR motif of the VSV L protein is essential for accurate stop–start transcription to synthesize full-length mRNAs and VSV growth in host cells (18).

NNS RNA viral L proteins were found to catalyze all enzymatic reactions required for viral mRNA synthesis and processing (capping, cap methylation, and polyadenylation) (11,16–17,19–27). L proteins share a general organization of putative domains with six highly conserved regions (called blocks I–VI) that are interrupted by variable sequences (28). The N-terminal region including block III and the C-terminal region including block VI were predicted to be RNA-dependent RNA polymerase (RdRp) (28) and *S*-adenosylmethionine-dependent MTase (29) domains, respectively. Conserved amino acid residues in the RdRp and MTase domains have been shown to be essential for transcription (21,30–31) and cap methylation (23–24,32–33), respectively. Furthermore, several conserved amino acid residues in block V of the VSV and CHPV L proteins have been identified as required for mRNA capping (16–17,34).

A VSV particle contains a transcriptionally active ribonucleoprotein complex, which consists of a genomic RNA wrapped with the nucleo- (N) proteins (called N-RNA) and the RdRp complex composed of the L protein and its co-factor phospho- (P) protein. The VSV RdRp complex initiates transcription at the 3′-end of the genomic RNA to produce the 47-nt uncapped leader RNA (35–37), and then sequentially synthesizes five mRNAs with the 5′-cap 1 structure and 3′-poly(A) tail from five tandemly aligned genes (3′-*N*-*P*-*M*-*G*-*L*-5′) by a stop–start mechanism (38–43). VSV pre-mRNA capping occurs at an early stage of mRNA chain elongation (42,44–46) and is required for efficient production of full-length mRNAs (18,34,47–48).

Using our *in vitro* reconstituted transcription system with the recombinant VSV L and P proteins and the native N-RNA template, cap-defective mutations in the HR motif of the L protein were found to impair mRNA synthesis, but not leader RNA synthesis, by causing aberrant stop–start transcription (18). After synthesis of the leader RNA, the cap-defective mutants frequently terminate N mRNA synthesis at positions +38 and +40 to synthesize transcripts initiated with 5′-ATP (called N1–38 and N1–40, respectively). Surprisingly, a part of the RdRp, stopped at the +38/+40 termination site, was found to generate an unusual 28-nt transcript from position +41 (N41–68) followed by a long 3′-polyadenylated transcript from position +157 (N157–1326, also called N_2_ RNA) initiated with non-canonical GTP. Higher rates of incorrect transcription termination and re-initiation using cryptic signals within the *N* gene resulted in dramatic attenuation of synthesis of downstream mRNAs as well as full-length N mRNA. Based on these observations, pre-mRNA capping with the PRNTase domain in the L protein during mRNA chain elongation was suggested to be a critical step to carry out accurate stop–start transcription leading to the production of full-length mRNAs.

In 2008, Li *et al*. (34) have identified the G1154, T1157, H1227 and R1228 residues in block V of the VSV L protein that are involved in cap formation using alanine scanning mutagenesis and our *in vitro* oligo-RNA capping assay with GTP as a substrate. Although our later study revealed that H1227 and R1228 are required for the L-pRNA intermediate formation in the PRNTase reaction, it still remains unknown which step(s) of capping (e.g. GTP hydrolysis, L-pRNA intermediate formation, pRNA transfer to GDP) is impaired by the G1154A and T1157A mutations. Furthermore, no other conserved amino acid residues in the putative PRNTase domain involved in mRNA capping have been identified.

Here, using amino acid sequences of more than 220 NNS RNA viral L proteins, available in a current public database, we found that five collinear sequence elements (called motifs A–E) are conserved in the putative PRNTase domains of NNS RNA viruses in the order *Mononegavirales* as suggested by our previous analysis of a small number of L proteins (13). By performing extensive mutagenesis of the VSV L protein, we identified key amino acid residues in these motifs as critical for the L-pRNA intermediate formation in the PRNTase reaction and accurate stop–start transcription for synthesis of full-length mRNAs. These results suggest that these motifs constitute the active site of the PRNTase domain, and pre-mRNA capping with the PRNTase domain plays an essential role in NNS RNA viral mRNA biogenesis.

## MATERIALS AND METHODS

### PRNTase and GTPase assays with the VSV L protein

The *in vitro* oligo-RNA capping assay was performed with the recombinant VSV L protein [60 ng, wild-type (WT) or mutant] using [α-^32^P]GDP and pppAACAG oligo-RNA as substrates according to the method described before (11,16,49). The L-pRNA intermediate formation assay was carried out with 0.3 μg of the recombinant VSV L protein (WT or mutant) and ^32^P-labeled pppAACAG oligo RNA as described previously (11,16,49). The GTPase assay was carried out with 0.3 μg of the recombinant VSV L protein (WT or mutant) using [γ-^32^P]GTP as described (15,16). WT and mutant L proteins were expressed in insect cells and purified as described previously (16,49).

### *In vitro* transcription assay with the VSV L protein

The *in vitro* VSV transcription assay was performed with the recombinant L protein (0.15 μg, WT or mutant), recombinant P protein (0.05 μg) and N-RNA complex (0.4 μg protein) as described previously (18,49). ^32^P-labeled and unlabeled VSV transcripts were synthesized with [α-^32^P]GTP and GTP, respectively (18,49). mRNAs were deadenylated with RNase H in the presence of oligo(dT) (49) when described in figure legends. Unlabeled VSV transcripts were post-labeled with vaccinia virus capping enzyme (Epicentre) in the presence of [α-^32^P]GTP as described (18), except in the presence of 0.1 mM *S*-adenosylmethionine instead of inorganic pyrophosphatase. ^32^P-labeled short (e.g. leader RNA) and long (e.g. mRNAs) transcripts were analyzed by electrophoresis in 20 and 5%, respectively, polyacrylamide gels containing 7 M urea (urea-PAGE) followed by autoradiography (18,49).

### Mini-genome assay

The VSV mini-genome assay using a plasmid expressing the VSV L protein (WT or mutant) was carried out as essentially described by Grdzelishvili *et al*. (23) with some modifications (see the Supplementary Methods). A reporter gene product and the VSV L protein expressed in the transfected cells were detected by an ELISA and Western blotting, respectively.

## RESULTS

### Generation of recombinant VSV L proteins with mutations in conserved amino acid residues surrounding the HR motif

**Figure 1.**
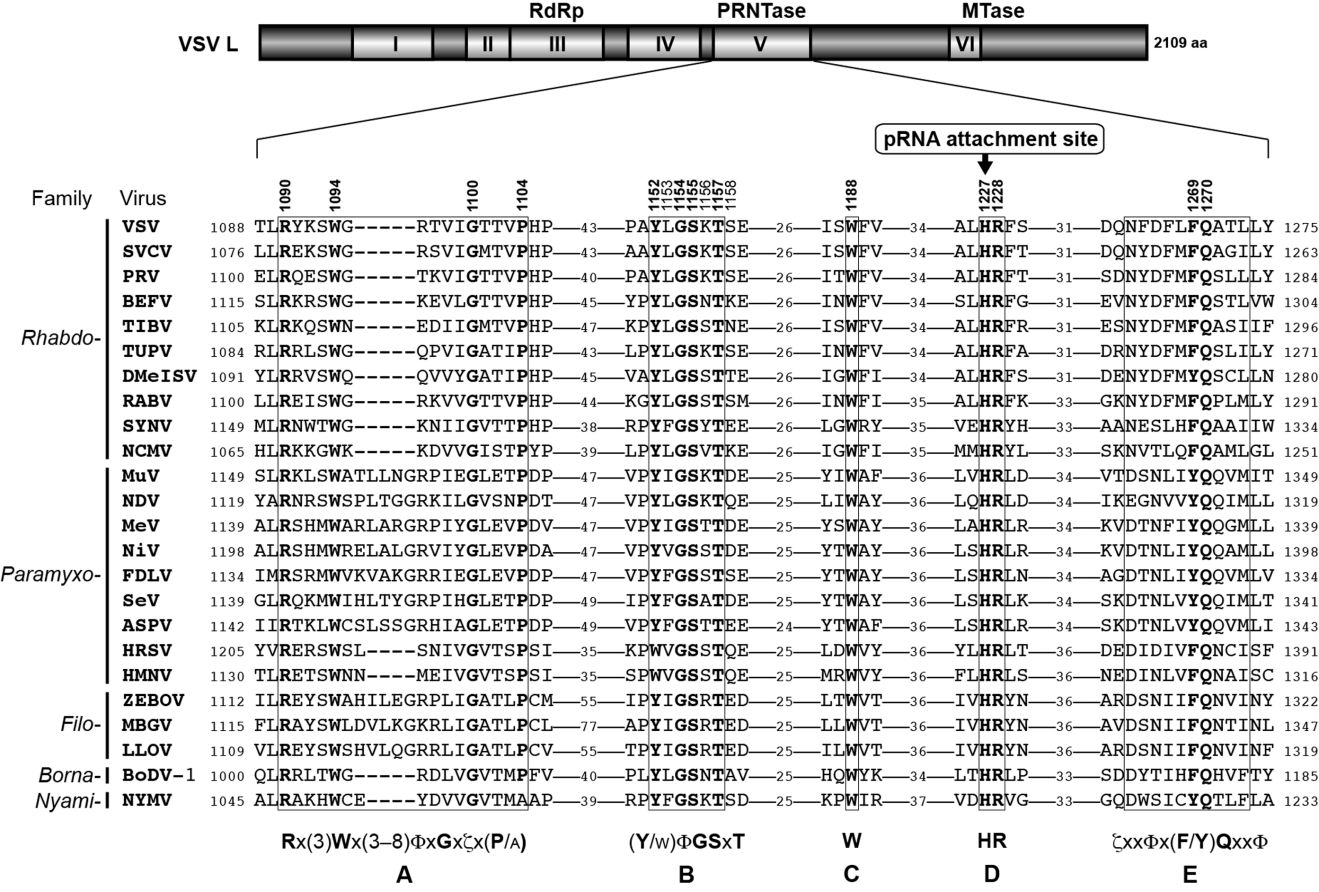
Conserved amino acid sequence motifs of the PRNTase domain in NNS RNA viral L proteins. A schematic structure of the L protein of VSV (*Vesiculovirus*) is shown with six conserved amino acid sequence blocks (I–VI) (28). RdRp, PRNTase and MTase indicate RNA-dependent RNA polymerase, GDP polyribonucleotidyltransferase and methyltransferase domains, respectively. The amino acid sequences of block V (PRNTase domain) of representative NNS RNA viral L proteins were aligned using the PSI-Coffee program (50) (see Supplementary Figure S1), and local sequences are shown with the numbers of intervening residues. Five consensus sequences are designated as motifs A–E (x, Φ and ζ indicate any, hydrophobic and hydrophilic amino acids, respectively). The numbers above the VSV L sequence show the positions of mutated amino acid residues in the VSV L protein. H1227 in motif D has been identified as a covalent pRNA attachment site (16). Virus names (virus genera) are as follows: SVCV, spring viremia of carp virus (*Sprivivirus*); PRV, perch rhabdovirus (*Perhabdovirus*); BEFV, bovine ephemeral fever virus (*Ephemerovirus*); TIBV, Tibrogargan virus (*Tibrovirus*); TUPV, Tupaia rhabdovirus (*Tupavirus*), DMelSV, *Drosophila melanogaster* sigma virus (*Sigmavirus*); RABV, rabies virus (*Lyssavirus*); SYNV, sonchus yellow net virus (*Nucleorhabdovirus*); NCMV, northern cereal mosaic virus (*Cytorhabdovirus*); MuV, mumps virus (*Rubulavirus*); NDV, Newcastle disease virus (*Avulavirus*); MeV, measles virus (*Morbillivirus*); NiV, Nipah virus (*Henipavirus*); FDLV, Fer-de-Lance virus (*Ferlavirus*); SeV, Sendai virus (*Respirovirus*); ASPV, Atlantic salmon paramyxovirus (*Aquaparamyxovirus*); HRSV, human respiratory syncytial virus (*Pneumovirus*); HMPV, human metapneumovirus (*Metapneumovirus*); ZEBOV, Zaire ebolavirus (*Ebolavirus*); MBGV, Marburg marburgvirus (*Marburgvirus*); LLOV, Lloviu virus (*Cuevavirus*); BoDV-1, Borna disease virus 1 (*Bornavirus*); NYMV, Nyamanini virus (*Nyavirus*). Their GenBank accession numbers are listed in Supplementary Table S1. The numbers on the left and right of the sequences indicate the amino acid positions in respective L proteins.

To identify highly conserved amino acid sequence motifs close to the catalytic HR motif as candidate motifs for the putative PRNTase domain, we analyzed amino acid sequences surrounding the HR motif (block V) from representative viruses belonging to different genera in the *Rhabdoviridae*, *Paramyxoviridae*, *Filoviridae*, *Bornaviridae* and *Nyamiviridae* families using the PSI-Coffee alignment program (50) (Supplementary Figure S1). As highlighted in Figure 1, we found that block V contains five conserved motifs, Rx(3)Wx(3–8)ΦxGxζx(P/A) (Φ and ζ indicate hydrophobic and hydrophilic amino acids, respectively; referred to as motif A), (Y/W)ΦGSxT (motif B), W (motif C), HR (motif D) and ζxxΦx(F/Y)QxxΦ (motif E). The G1154 and T1157 residues of the VSV L protein are present within motif B that is located ∼75 residues upstream of motif D. We also confirmed that these motifs are strikingly conserved in L proteins of more than 220 known NNS RNA viruses (see Supplementary Table S1 and Figure S2).

**Figure 2.**
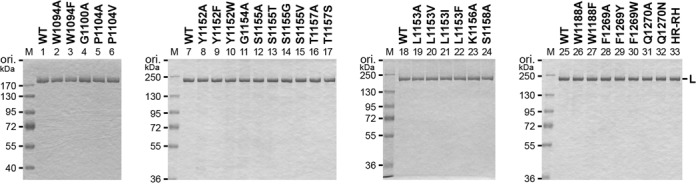
Sodium dodecylsulphate-polyacrylamide gel electrophoresis (SDS-PAGE) analysis of recombinant wild-type (WT) and mutant VSV L proteins. The WT and mutant VSV L proteins (1 μg) were analyzed by 7.5% SDS-PAGE followed by Coomassie Brilliant Blue staining. M lanes show marker proteins with the indicated molecular masses. The names of the point mutants contain the original amino acid (one-letter code) at the indicated position in the VSV L protein followed by the replacement amino acid. The HR-RH mutant carrying the H1227R and R1228H mutations (lane 33) is a representative of the previously identified cap-defective mutants (16).

To analyze whether these conserved and some semi-conserved amino acid residues of the VSV L protein participate in mRNA capping reactions, we mutated them to alanine and/or closely related amino acids (see Figure 2). We divided mutants into four groups, and expressed and purified them together with the WT L protein. Their purity was verified by sodium dodecylsulphate-polyacrylamide gel electrophoresis followed by staining with Coomassie Brilliant Blue (Figure 2). It should be noted that, since R1090A and R1090K mutants were not obtained in high qualities and quantities due to their extremely low expression levels in insect cells (not shown), these mutants could not be further characterized. In addition, since the solubility of G1154A as well as G1154S (not shown) were significantly lower than those of WT and other mutant L proteins, the G1154 mutants were solubilized in the presence of 1 M NaCl instead of 0.3 M NaCl, which was used for the WT and other mutants. These observations suggest that mutations in R1090 and G1154 might affect the structural integrity of the domain or the whole protein, leading to their lower expression and solubility, respectively.

### Conserved amino acid sequence motifs surrounding the HR motif are required for the PRNTase activity

**Figure 3.**
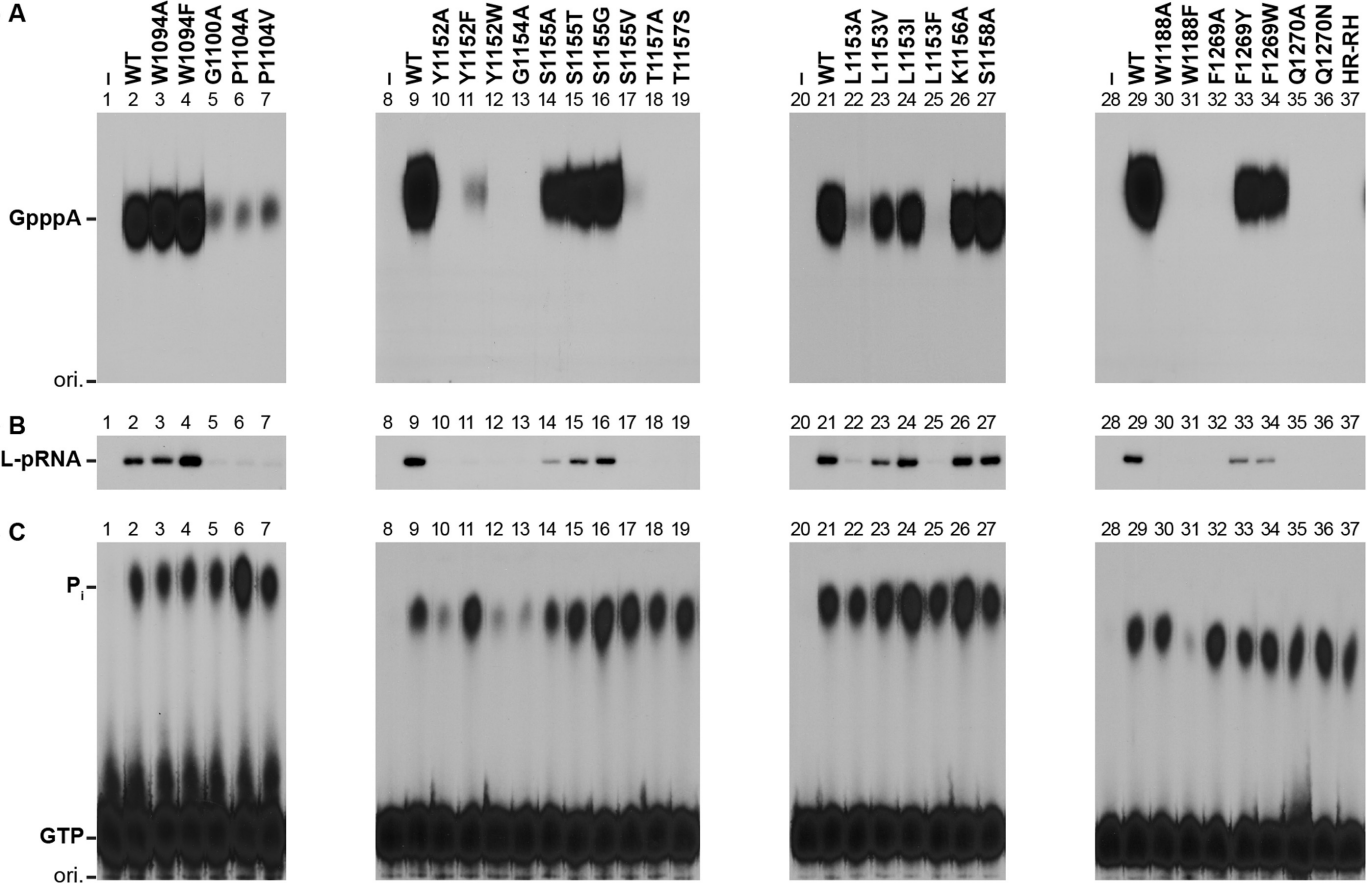
Conserved amino acid residues in the PRNTase domain of the VSV L protein are required for the L-pRNA intermediate formation in RNA capping. (**A**) The WT and mutant VSV L proteins (60 ng) were subjected to *in vitro* capping reactions with pppAACAG and [α-^32^P]GDP. Digests of RNA products with nuclease P1 were analyzed by PEI-cellulose TLC followed by autoradiography. The positions of the origin (ori.) and standard GpppA cap analogue are shown. (**B**) The WT and mutant VSV L proteins (0.3 μg) were incubated with pppAACAG (labeled with [α-^32^P]AMP). The resulting L-pRNA intermediate was analyzed by 7.5% SDS-PAGE followed by autoradiography. (**C**) The WT and mutant VSV L proteins (0.3 μg) were incubated with [γ-^32^P]GTP. Released ^32^P_i_ was analyzed by PEI-cellulose TLC followed by autoradiography. Lanes 1, 8, 20 and 28 indicate no L protein.

First, we subjected the highly purified L mutants to the *in vitro* cap formation assay with pppAACAG and [α-^32^P]GDP to measure their PRNTase activities independently of their GTPase activities (11,16,49) (Figure 3A and Table 1). We used the HR-RH mutant (lane 37), which possesses an RH sequence instead of the HR motif (motif D), as a representative of the previously characterized cap-defective L mutants (16). The cap formation activities of W1094A (lane 3) and W1094F (lane 4) were equivalent or higher than that of the WT L protein (lane 2), whereas the G1100A (lane 5), P1104A (lane 6) and P1104V (lane 7) mutations markedly reduced the cap formation activity to 11–15% of the WT activity. The Y1152A (lane 10) and Y1152W (lane 12) mutants were completely inert, whereas Y1152F (lane 11) showed about 4% of the WT activity. G1154A (lane 13) and G1154S (not shown) did not show any cap formation activity. While the S1155A (lane 14), S1155T (lane 15) and S1155G (lane 16) mutants showed 20–53% of the WT activity, S1155V (lane 17) exhibit an extremely low activity (∼2%), indicating that this residue could be partially replaced with a small amino acid with lower hydrophobicity (G > T > A). The T1157A (lane 18) and T1157S (lane 19) mutants were completely inactive. The L1153A (lane 22) and L1153F (lane 25) mutants showed low cap formation activities (10 and 3%, respectively, of the WT activity), whereas the L1153V and L1153I mutants exhibited modest activities (39 and 45%, respectively), indicating that larger aliphatic amino acids (I > V) could be partially substituted for L1153. On the other hand, alanine substitutions of non-conserved K1156 (lane 26) and S1158 (lane 27) did not have significant effects on the cap formation activity. The W1188A mutation also abolished the cap formation activity (lane 30), but this residue could not be replaced with other aromatic amino acids, such as F (lane 31), Y (not shown) and H (not shown). Interestingly, although F1269A (lane 32) was inactive in the cap formation, F1269Y (lane 33) and F1269W (lane 34) exhibited 31 and 18% of the WT activity, respectively. Furthermore, similar to the HR-RH mutant (lane 37), Q1270A (lane 35), Q1270N (lane 36) and Q1270E (not shown) displayed no activity.

**Table 1.** Relative enzymatic activities of mutant L proteins

Mutant	Relative activity (% of WT)^a^				
	PRNTase		GTPase	RdRp	
	GpppA	L-pRNA	P_i_	Leader RNA	mRNAs
W1094A	136 ± 5	96 ± 4	86 ± 6	33 ± 6	17 ± 3
W1094F	181 ± 10	239 ± 32	102 ± 11	98 ± 8	88 ± 17
G1100A	11 ± 1	10 ± 3	99 ± 12	128 ± 11	6 ± 2
P1104A	12 ± 1	13 ± 2	242 ± 26	27 ± 3	14 ± 1
P1104V	15 ± 0	10 ± 1	124 ± 17	84 ± 8	39 ± 4
Y1152A	0 ± 0	3 ± 3	42 ± 4	0 ± 0	0 ± 0
Y1152F	4 ± 0	9 ± 9	161 ± 20	49 ± 3	30 ± 0
Y1152W	0 ± 0	3 ± 2	42 ± 6	1 ± 0	0 ± 0
G1154A	0 ± 0	3 ± 3	35 ± 1	0 ± 1	0 ± 0
S1155A	20 ± 1	20 ± 1	90 ± 4	56 ± 8	34 ± 4
S1155T	44 ± 4	40 ± 4	142 ± 9	30 ± 3	20 ± 7
S1155G	53 ± 3	68 ± 3	244 ± 11	38 ± 6	30 ± 6
S1155V	2 ± 0	3 ± 3	187 ± 15	4 ± 0	0 ± 0
T1157A	0 ± 0	1 ± 1	127 ± 1	80 ± 7	2 ± 0
T1157S	0 ± 0	1 ± 1	159 ± 1	66 ± 5	3 ± 0
L1153A	10 ± 5	12 ± 3	83 ± 4	0 ± 2	0 ± 0
L1153V	39 ± 3	48 ± 2	124 ± 17	3 ± 1	3 ± 1
L1153I	45 ± 15	98 ± 1	187 ± 23	9 ± 3	15 ± 3
L1153F	3 ± 0	5 ± 2	96 ± 14	1 ± 1	3 ± 2
K1156A	81 ± 6	104 ± 1	187 ± 26	109 ± 11	122 ± 24
S1158A	88 ± 10	87 ± 1	111 ± 26	119 ± 17	48 ± 10
W1188A	0 ± 0	1 ± 1	102 ± 3	4 ± 1	0 ± 0
W1188F	0 ± 0	2 ± 2	16 ± 2	13 ± 2	1 ± 0
F1269A	0 ± 0	3 ± 3	163 ± 5	38 ± 2	3 ± 0
F1269Y	31 ± 6	35 ± 8	107 ± 7	80 ± 6	85 ± 4
F1269W	18 ± 2	21 ± 0	130 ± 5	114 ± 2	37 ± 4
Q1270A	0 ± 0	2 ± 2	108 ± 11	102 ± 4	5 ± 0
Q1270N	0 ± 0	1 ± 1	162 ± 19	16 ± 1	1 ± 0
HR-RH	0 ± 0	3 ± 3	92 ± 5	101 ± 7	4 ± 0

^a^The PRNTase, GTPase and RdRp activities of the mutant L proteins were measured by the *in vitro* oligo-RNA capping, L-pRNA intermediate formation, GTPase and transcription assays as shown in Figures 3 and 4. Relative enzymatic activities of the mutant L proteins were expressed as percentages of the WT activities. Data represent the means and standard deviations from three independent experiments.

To investigate which step(s) of the PRNTase reaction is abrogated with the mutations, we analyzed effects of these mutations on the covalent L-pRNA intermediate formation, a critical step of the cap formation (Figure 3B and Table 1). We found that relative L-pRNA intermediate formation activities of the cap-defective mutants [G1100A (lane 5), P1104A/V (lanes 6 and 7), Y1152A/F/W (lanes 10–12), G1154A (lane 13), S1155V (lane 17), T1157A/S (lanes 18 and 19), L1153A/F (lanes 22 and 25), W1188A/F (lanes 30 and 31), F1269A (lane 32) and Q1270A/N (lanes 35 and 36)] were consistent with their cap formation activities (Figure 3A and Table 1), suggesting that these mutations diminished or abolished the PRNTase activity in the step of the L-pRNA intermediate formation. However, we did not find any mutations that affect the pRNA transfer reaction, while not affecting the intermediate formation reaction.

We also examined the effects of these mutations on the GTPase activity of the L protein (Figure 3C and Table 1), which releases the γ-phosphate of GTP as inorganic phosphate (P_i_). Any mutations of the G1100 (lane 5), P1104 (lanes 6 and 7), S1155 (lanes 14–17), T1157 (lanes 18 and 19), L1153 (lanes 22–25), F1269 (lanes 32–34) and Q1270 (lanes 35 and 36) residues did not abolish the GTPase activity, suggesting that these residues are specifically required for the PRNTase activity. In contrast, the Y1152A (lane 10), Y1152W (lane 12), G1154A (lane 13) and W1188F (lane 31) mutations reduced the GTPase activity, indicating that these mutations affect both the GTPase and PRNTase activities.

### Cap-defective mutants produce uncapped abortive transcripts by aberrant stop–start transcription

**Figure 4.**
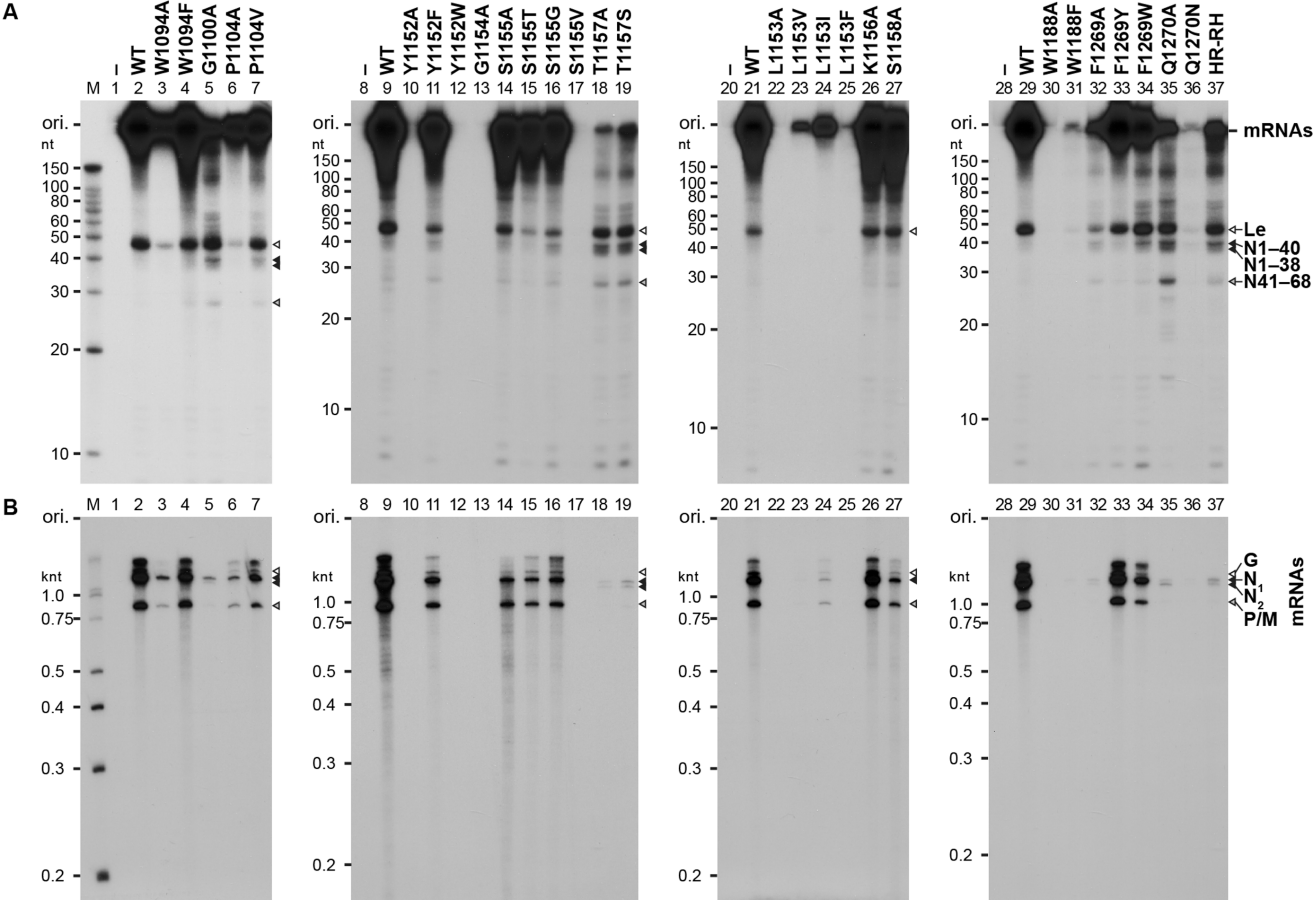
Cap-defective mutant L proteins are not able to synthesize full-length mRNAs efficiently. The WT and mutant VSV L proteins (0.15 μg) were subjected to *in vitro* transcription reactions with [α-^32^P]GTP, the other three NTPs, the recombinant P protein (0.05 μg) and the native N-RNA complex (0.4 μg). After treatment with RNase H and oligo(dT), transcripts were analyzed by 20% (**A**) or 5% (**B**) urea-PAGE followed by autoradiography. Lanes 1, 8, 20 and 28 indicate no L protein. M lanes show marker RNAs with the indicated lengths. The positions of previously identified transcripts (18) are indicated on the right. Le indicates the leader RNA.

Our previous study (18) showed that the cap-defective mutations in the HR motif (motif D) negatively impact mRNA synthesis, but not leader RNA synthesis. To analyze the effects of the mutations in other conserved motifs on the transcription activity of the L protein, we reconstituted the transcription reaction with the WT or mutant L protein, the recombinant P protein and the N-RNA template. After the reactions, poly(A) tails on mRNAs were digested with RNase H in the presence of oligo(dT). Short (e.g. leader RNA) and long (e.g. deadenylated mRNAs) transcripts were analyzed by 20% (Figure 4A and Table 1) and 5% (Figure 4B and Table 1), respectively, urea-PAGE. Similar to the motif D mutants, such as HR-RH (lane 37) (18), G1100A (lane 5), T1157A (lane 18), T1157S (lane 19) and Q1270A (lane 35) showed 66–128% of the WT activity in leader RNA synthesis, but displayed 2–6% of the WT activity in mRNA synthesis. Furthermore, these cap-defective mutants synthesized large amounts of short abortive transcripts, which co-migrate with previously-identified N1–40 (40 nt), N1–38 (38 nt) and N41–68 (28 nt) RNAs (Figure 4A, lanes 5, 18, 19 and 35), as well as small amounts of 1.3- and 1.2-knt RNAs, which co-migrate with the N_1_ (full-length N mRNA, N1–1326) and N_2_ (N157–1326) RNAs, respectively (Figure 4B, lanes 5, 18, 19 and 35). Although W1188F (lane 31), F1269A (lane 32) and Q1270N (lane 36) exhibited weaker RNA synthesis activities than those of other cap-defective mutants, their mRNA synthesis activities (1–3% of the WT activity) were significantly lower than their leader RNA synthesis activities (13–38% of the WT activity). In contrast, W1094A (lane 3), P1104A (lane 6), Y1152A (lane10), Y1152W (lane 12), G1154A (lane 13), S1155V (lane 17), L1153A/V/I/F (lanes 22–25) and W1188A (lane 30) showed low or no activities to synthesize both the leader RNA and mRNAs. Other mutations had no or moderate negative effects on synthesis of both the leader RNA and mRNAs. We also found that W1094 (lane 4), Y1152 (lane 11) and F1269 (lanes 33 and 34) could be functionally replaced with another aromatic amino acid(s) in transcription.

**Figure 5.**
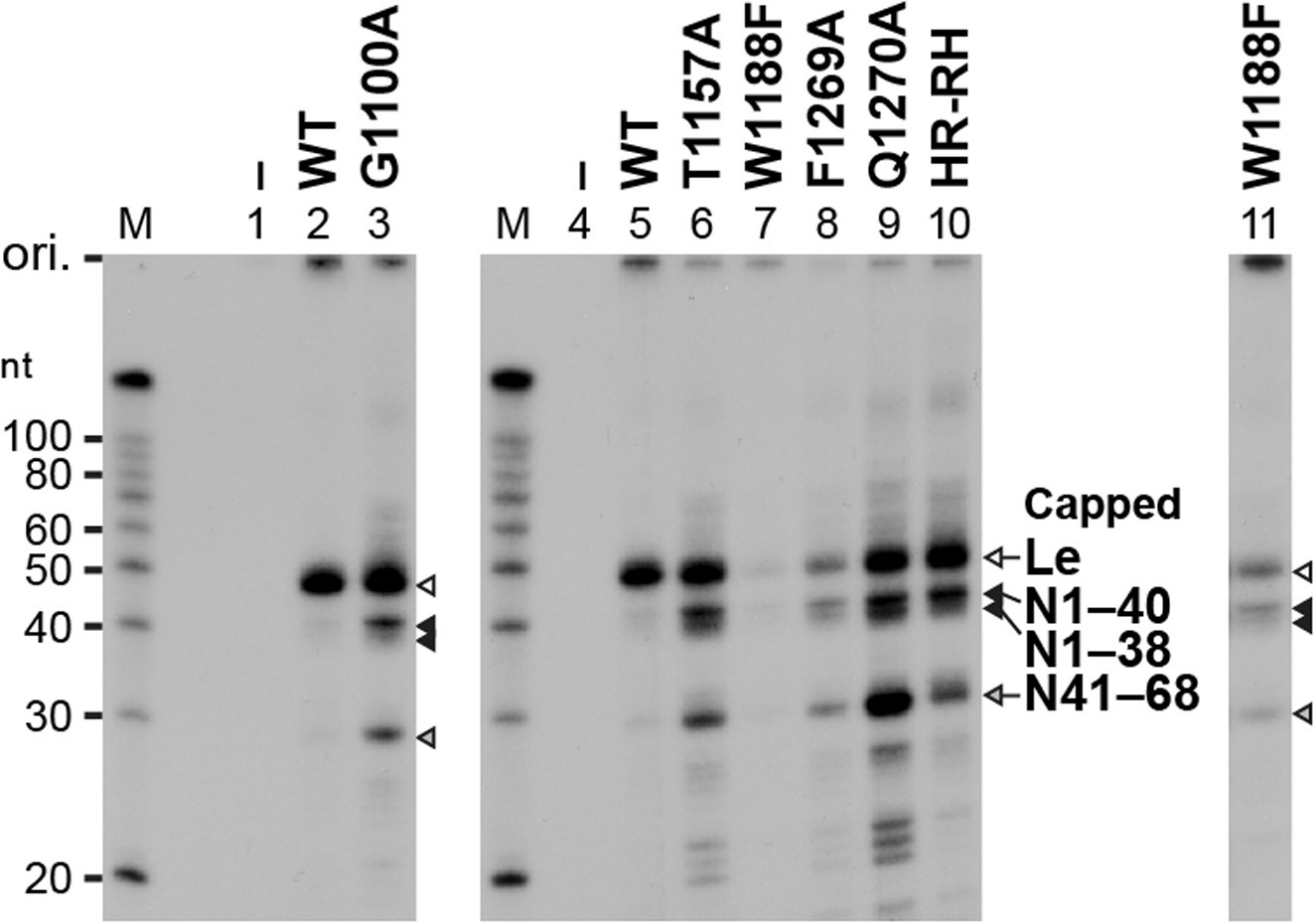
Cap-defective mutant L proteins produce uncapped abortive short transcripts. Transcripts, synthesized by the WT or cap-defective mutant L proteins, were post-labeled by vaccinia virus capping enzyme in the presence of [α-^32^P]GTP and analyzed by 20% urea-PAGE followed by autoradiography. Lanes 1 and 4 indicate no L protein. Lane 11 indicates a longer exposure of lane 7. The positions of previously identified capped RNAs (18) are shown.

To confirm whether 40, 38 and 28-nt RNAs produced with the selected cap-defective mutants (G1100A, T1157A, W1188F, F1269A and Q1270A) have 5′-tri- or di-phosphate ends, these RNAs were capped with vaccinia virus capping enzyme in the presence of [α-^32^P]GTP (Figure 5). As reported previously (18), the WT L protein synthesized the leader RNA as a major substrate for vaccinia virus capping enzyme (lanes 2 and 5), whereas the HR-RH cap-defective mutant produced N1–40, N1–38 and N41–68 RNAs, in addition to the leader RNA, as the substrates (lane 10). Similarly, the short transcripts (leader RNA and RNAs with 40, 38 and 28 nt) synthesized by G1100A (lane 3), T1157A (lane 6), F1269A (lane 8) and Q1270A (lane 9) were capped with vaccinia virus capping enzyme. A longer exposure of lane 7 to X-ray film showed that W1188F also produces the uncapped 40, 38 and 28-nt RNAs in addition to the leader RNA (lane 11). From these results, we suggest that the cap-defective G1100A, T1157A, W1188F, F1269A and Q1270A mutants produced short 5′-triphosphorylated N1–40, N1–38 and N41–68 RNAs by aberrant stop–start transcription, as reported for the cap-defective motif D mutants (e.g. H1277R, R1288H, HR-RH) (18).

**Figure 6.**
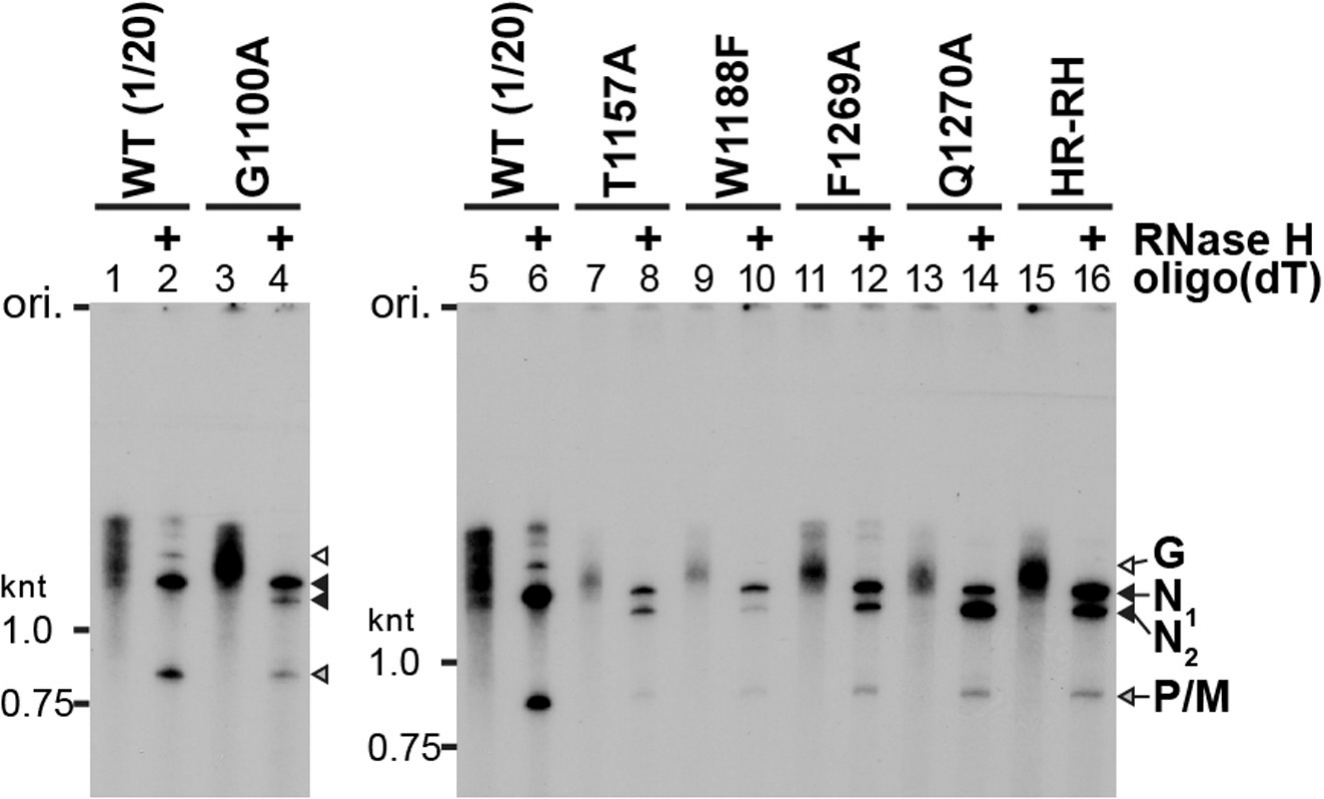
The cap-defective mutant L proteins synthesize small amounts of polyadenylated full-length (N_1_) and 5′-truncated (N_2_) N mRNA. ^32^P-Labeled transcripts, synthesized by the WT or mutant L protein, were treated with or without RNase H and oligo(dT) and analyzed by 5% urea-PAGE followed by autoradiography. The sample volume of transcripts synthesized with the WT L protein (lanes 1, 2, 5 and 6) was 20-fold smaller than those of transcripts synthesized with the other mutants.

Furthermore, we analyzed the polyadenylation status of the long transcripts synthesized by the selected cap-defective mutants (G1100A, T1157A, W1188F, F1269A and Q1270A) (Figure 6). As reported before (18), mRNAs produced by the WT L protein migrated as a broad smear (lanes 1 and 5), whereas mRNAs deadenylated with RNase H and oligo(dT) were separated into three discrete bands of 1.3-knt N, 0.8-knt P/M and 1.7-knt G mRNAs (lanes 2 and 6). The long RNAs with heterogeneity in length, synthesized by the G1100A (lane 3), T1157A (lane 7), W1188F (lane 9), F1269A (lane 11) and Q1270A (lane 13) cap-defective mutants, were deadenylated with RNase H into 1.3-knt, 1.2-knt and 0.8-knt RNAs (lanes 4, 8, 10, 12 and 14), which co-migrate with deadenylated N_1_ (full-length N mRNA, N1–1326), N_2_ (N157–1326), and P mRNAs, respectively, synthesized by the HR-RH mutant (lane 16) (18). Production of polyadenylated N_2_ RNA as well as the short abortive RNAs (e.g. N1–40, N41–68) by aberrant stop–start transcription is one of the characteristic futures of the cap-defective motif D mutants. Thus, we conclude that the G1100, T1157, W1188, F1269 and Q1270 residues are also required for co-transcriptional mRNA capping and efficient synthesis of full-length mRNAs.

### The PRNTase motifs are required for VSV gene expression in host cells

**Figure 7.**
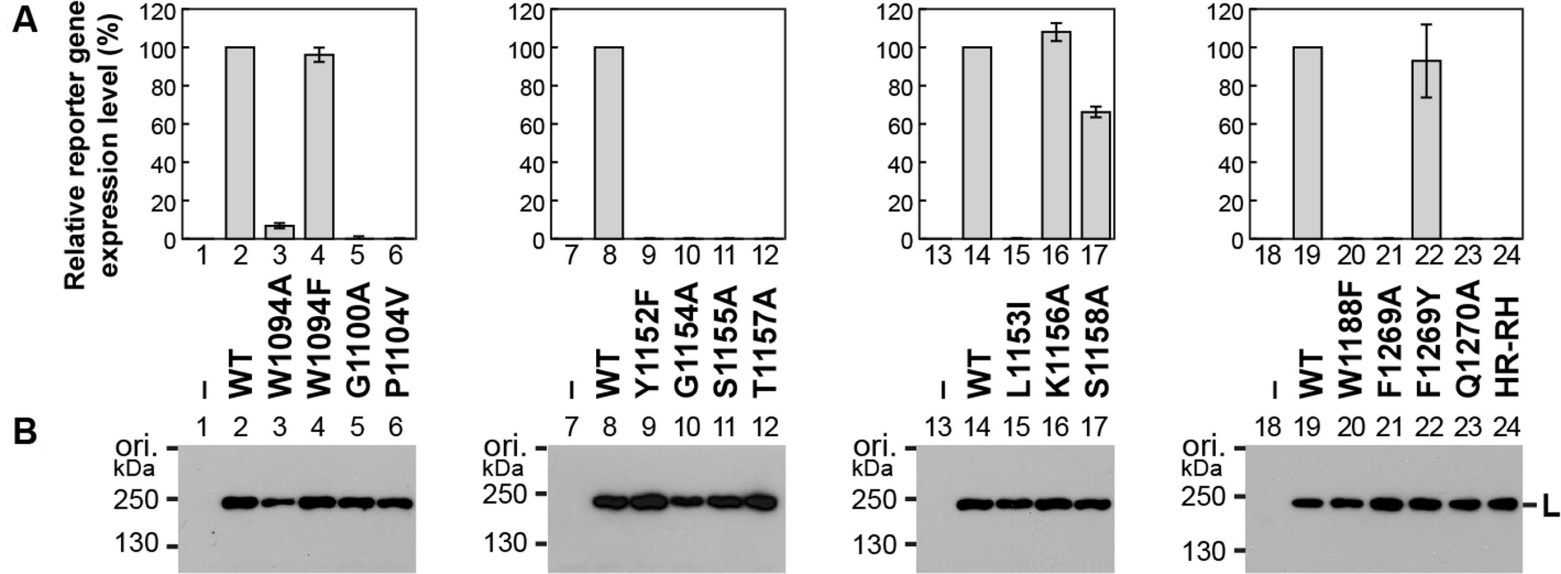
The cap-defective mutations in the VSV L protein abolish gene expression from a mini-genome in host cells. The VSV mini-genome reporter assay (23) was performed with plasmids expressing the N, P and L (WT or mutant) proteins. (**A**) The relative expression levels of a reporter gene product in cells expressing the WT (defined as 100%) or mutant L protein are shown. Columns and error bars represent the means and standard deviations, respectively, from three independent experiments. Columns 1, 7, 13 and 18 indicate no plasmid expressing the L protein. (**B**) The WT and mutant L proteins expressed in the cells were detected by Western blotting. The results shown are representatives of the three independent experiments.

To analyze effects of mutations in the PRNTase motifs on VSV gene expression in host cells, we performed a mini-genome assay with a plasmid expressing a negative strand genome with a reporter gene, instead of the five VSV genes (23). In the presence of the N, P and L proteins expressed from supporting plasmids, the mini-genome is replicated and transcribed into reporter mRNA. The expression levels of the reporter gene product in cells expressing selected mutant L proteins were compared with that in cells expressing the WT L protein (Figure 7A). We also confirmed that the mutant L proteins were expressed at levels similar to that of the WT protein in the transfected cells (Figure 7B). Consistent with the *in vitro* mRNA synthesis activities of W1094A and W1094F (Figure 4B), cells expressing these mutants showed 7% (Figure 7A, column 3) and 96% (column 4), respectively, of the reporter gene expression level in cells expressing the WT L protein. Gene expression from the mini-genome was not observed in cells expressing the cap-defective mutants, G1100A (column 5), T1157A (column 12), W1188F (column 20), F1269A (column 21), Q1270A (column 23) and HR-RH (column 24) as well as the transcription-defective mutants, Y1152A (not shown) and G1154A (column 10). Unexpectedly, P1104V (column 6), Y1152F (column 9), S1155A (column 11) and L1153I (column 15) did not support gene expression from the mini-genome, although they retained weak transcription activities *in vitro*, suggesting essential roles of P1104, Y1152, S1155 and L1153 in VSV gene expression in host cells. Consistent with *in vitro* results (Figures 3 and 4), F1269 could be functionally replaced with other aromatic amino acids, Y (column 22) and W (not shown).

As we reported for the motif D mutations (18), recombinant VSVs with cap-defective mutations (e.g. T1157A, W1188F, F1269A, Q1270A) could not be generated from cDNAs using the reverse genetics system (data not shown). In contrast, recombinant VSVs with F1269Y and F1269W were rescued from cDNAs. Consistent with the *in vitro* transcription activities of F1269Y and F1269W (Figure 4), VSV with F1269Y formed plaques similar to WT VSV, while VSV with F1269W formed smaller plaques (Supplementary Figure S3). Taken together, we conclude that cap-defective mutations in the L protein cause defects in the VSV gene expression at the step of production of translatable mRNAs, thereby being lethal to the virus.

## DISCUSSION

In addition to the catalytic amino acid residues (H1227 and R1228) in motif D (16), we identified other conserved amino acid residues in motifs A (G1100), B (T1157), C (W1188) and E (F1269 and Q1270) of the putative PRNTase domain of the VSV L protein that are essential or important for the L-pRNA intermediate formation and synthesis of full-length mRNAs, but not for GTP hydrolysis or synthesis of the uncapped leader RNA. In contrast, conserved residues in the N-terminal region of the VSV L protein [e.g. H360, H639, D714 (RdRp active site)] were found to be required for synthesis of both the leader RNA and mRNAs, but not for any steps of mRNA capping (16,18). These PRNTase motifs are strikingly conserved in L proteins of divergent NNS RNA viruses belonging to the different families, *Rhabdoviridae* (e.g. rabies), *Paramyxoviridae* (e.g. measles, Nipah, respiratory syncytial), *Filoviridae* (e.g. Ebola), *Bornaviridae* (e.g. Borna disease) and *Nyamiviridae* (e.g. Nyamanini), in the order *Mononegavirales* and rhabdovirus-like bipartite negative strand RNA viruses (see Supplementary Table S1, Figures S2 and S4, and Supplementary Discussion), suggesting that they play common roles in substrate binding, catalysis and/or structural maintenance of the PRNTase domain.

**Figure 8.**
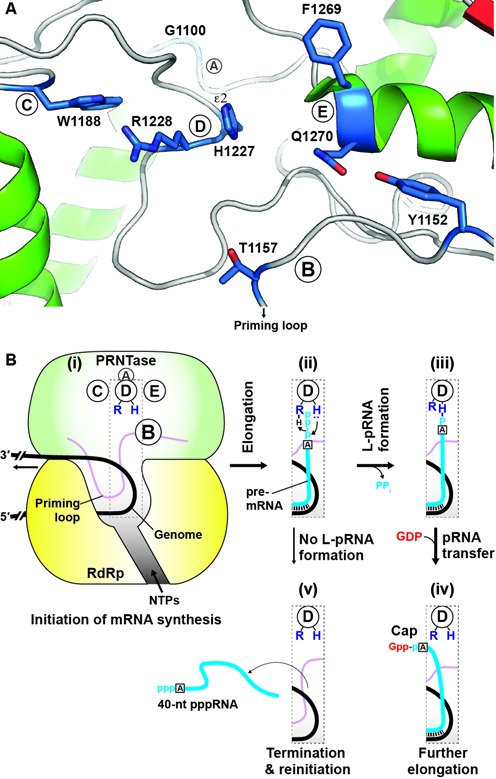
Localization of the amino acid residues required for the L-pRNA intermediate formation in the putative PRNTase domain and model of co-transcriptional mRNA capping. (**A**) A ribbon diagram of a three-dimensional structure of the PRNTase active site was generated using the atomic coordinates for the cryo-EM structure of the VSV L protein (PDB: 5A22) and the PyMOL software (http://www.pymol.org). The amino acid residues, required for the L-pRNA intermediate formation, in PRNTase motifs A–E are shown as stick models. The indicated *N*^ϵ2^ position of the catalytic histidine residue (H1227) in motif D is the covalent pRNA attachment site (16). The hydroxyl group of Y1152 in motif B is hydrogen-bonded to the side-chain carbonyl group of Q1270 in motif E. (**B**) A schematic structure of the VSV L protein is depicted only with the RdRp and PRNTase domains. After *de novo* initiation of mRNA synthesis from an internal promoter in the genome (i), the 5′-end of triphosphorylated pre-mRNA (pppA-RNA) is extruded from the transcribing RdRp domain at an early stage of mRNA chain elongation (ii) to gain access to the active site of the PRNTase domain. Immediately after the formation of the covalent L-pRNA formation (iii) with the catalytic amino acid residues (histidine and arginine) in motif D (16), pRNA is transferred to GDP to form capped pre-mRNA (Gpp-pA-RNA), which is further elongated to full-length mRNA with the RdRp domain (iv). If the PRNTase domain fails to form the L-pRNA intermediate, the RdRp domain releases 40-nt pppRNA and reinitiates transcription using a cryptic initiation signal (v).

A very recent cryo-electron microscopic (EM) analysis of the VSV L protein complexed with a fragment of the P protein produced a high-resolution density map of the complex leading to an atomic model of almost the entire L protein structure, composed of the N-terminal RdRp domain with blocks I to III, capping domain with blocks IV and V, connector domain, MTase domain with block VI and the C-terminal domain (51). As shown in Figure 8A and Supplementary Figure S5, the amino acid residues required for the L-pRNA intermediate formation are localized in close proximity to H1227 in the C-terminal part (block V, putative PRNTase domain) of the capping domain, juxtaposing an RNA exit channel of the RdRp domain. These observations suggest that all these residues constitute the active site of the PRNTase domain waiting for 5′-ends of pre-mRNAs that emerge from the exit channel.

In the cryo-EM structure of the non-transcribing VSV L protein (51), G1100 in motif A is located on a loop between two α-helices and is within ∼12 Å distance from H1227 in motif D (Figure 8A and Supplementary Figure S5). This highly conserved G residue was suggested to be required for the efficient intermediate formation possibly by playing a structural role in maintaining the loop or interacting with pppRNA via its backbone amide group.

Motif B is located in the middle position of a large loop structure (residues 1136–1173) between a β-sheet and α-helix (51). Interestingly, a C-terminal portion (residues 1157–1173) of this loop is deeply inserted into the active site cavity of the RdRp domain, suggesting that it serves as a priming loop for *de novo* transcription initiation (51). Thus, it is not surprising that amino acid residues in this loop are involved in capping and/or transcription.

Although L proteins of paramyxoviruses belonging to the *Pneumovirinae* subfamily [e.g. human respiratory syncytial virus (HRSV), human metapneumovirus] contain W instead of Y in motif B, W could not be substituted for Y1152 of the VSV L protein in all the enzymatic reactions. In contrast, we found that Y1152F exhibits weak capping and transcription activities, suggesting that an aromatic phenyl group (Y > F), but not indolyl group, is necessary for function at this position in the VSV L protein. In the cryo-EM structure (51), the hydroxyl group of Y1152 is hydrogen-bonded to the side-chain carbonyl group of Q1270 in motif E, suggesting that the hydroxyl group plays an important role in bringing motif B in close proximity to motif D (Figure 8A and Supplementary Figure S5).

Since T1157 in motif B could not be functionally replaced with A or S in the PRNTase reaction (Figure 3), both the β-hydroxyl and methyl groups of T1157 seem to be essential for the intermediate formation. Our previous study (16) proposed that a lone pair of electrons on the ϵ2-nitrogen atom of the H1227 residue in motif D nucleophilically attacks the 5′-α-phosphorus of pppRNA with the help of R1228. In the cryo-EM structure of the VSV L protein (51), T1157 was found to be located in the vicinity (∼10 Å) of the catalytic H1227 (motif D) in a large loop between two α-helices. Therefore, one possibility is that, similar to the S or T residue in the glycine-rich phosphate binding (P-) loop [GxxxxGK(S/T)] and P-loop like motifs in nucleotide binding proteins (52,53), the β-hydroxyl group of T1157 in motif B may contact a phosphate oxygen(s)/hydrogen(s) in pppRNA directly or via a metal ion during the intermediate formation with the adjacent H1227 residue.

In the EM structure of the VSV L protein (51), W1188 (motif C) is present on a loop between two α-helices, and is in close proximity (∼8 Å) to H1227 in motif D (Figure 8A and Supplementary Figure S5). The cap-defective W1188F mutant, but not W1188A, retained a low transcription activity and exhibited aberrant stop–start transcription, suggesting that F, an aromatic amino acid, could be substituted for W in partially catalyzing RNA synthesis, but not capping. Interestingly, the W1188F mutant showed a GTPase activity that is significantly lower than those of the WT and W1188A L proteins, indicating that a phenyl group at this position impairs the GTPase reaction, which may occur in the vicinity of this residue.

F1269 and Q1270 in motif E are located at an N-terminal end of an α-helix in close proximity (∼7–8 Å) to H1227 in the cryo-EM structure of the VSV L protein (51) (Figure 8A and Supplementary Figure S5). Consistent with conservation of an aromatic amino acid (F, Y or H) in motif E (Supplementary Table S1, Figures S2), F1269 could be functionally replaced with another aromatic amino acid (Y or W). Since aromatic side chains in many nucleic acid binding proteins are known to be involved in nucleic acid binding via base stacking interactions (54), the aromatic side chain in motif E could play an essential role in RNA binding during the L-pRNA intermediate formation. The Q residue in motif E was also suggested to be required for RNA binding, because its amide group has the potential to recognize RNA (e.g. nucleotide bases) via hydrogen bonding (55,56). Furthermore, the hydrogen bonding of Q1270 with Y1152 in motif B suggests its structural role in forming the PRNTase active site as described above.

We have not yet found any specific amino acid residues required for pRNA transfer to GDP (e.g. GDP binding residues), but not for the intermediate formation. We predict that some of amino acid residues required for binding to the β-γ phosphates of pppRNA and the leaving PP_i_ may be involved in interactions with the α-β phosphates of GDP after the formation of the L-pRNA intermediate. We propose that R1228 next to H1227 may bind to the β-γ phosphate oxyanions of pppRNA via ionic interactions and/or serve as a proton donor (general acid) to facilitate the PP_i_ release. R1228 could be functionally replaced with H although to a lesser extent, but not with K (16), suggesting that the secondary amine at the ϵ position in the positively charged guanidino group of R1228 is one of the chemical groups essential for the intermediate formation and possibly pRNA transfer. Although Liang *et al*. (51) described that G1154 and T1157 are involved in guanosine nucleotide binding, there is no experimental evidence to support this hypothesis.

Some conserved residues in motifs A–E may interact with common elements in 5′-ends of NNS RNA viral pre-mRNA, such as 5′-triphosphate, the first purine base (A or G), riboses and internal phosphates. Since both motifs B and D are present on the long flexible loops in the non-liganded domain, the structure of the active site may undergo some structural re-organization upon pppRNA binding followed by intermediate formation. On the other hand, since mRNAs of NNS RNA viruses belonging to different genera/families contain unique sets of mRNA-start sequences, amino acid residues of L proteins conserved in respective genera/families will likely be involved in sequence-specific recognition of mRNA 5′-end sequences (see Supplementary Discussion).

The newly identified mutations, conferring the defect in the L-pRNA intermediate formation, induced termination of N mRNA synthesis mainly at position +40 (Figures 4 and 5), as previously reported for the mutations in motif D (18). We suggest that the L-pRNA intermediate formation with the PRNTase domain of the L protein is a key event that controls the fate of the RdRp domain at an early stage of mRNA chain elongation (Figure 8B). Since the minimum lengths of capped VSV transcripts naturally or artificially terminated during *in vitro* transcription were reported to be 23–37 nt (44–46), the L-pRNA intermediate formation may occur immediately before the cap formation on nascent pre-mRNAs. If the L protein is not able to form the covalent L-pRNA intermediate, the RdRp domain in the L protein may terminate transcription at position +40 to release uncapped transcripts before the transition into further elongation of mRNA chain. Interestingly, a small-molecule capping inhibitor, possibly interacting with regions adjacent to motifs B and E in the HRSV L protein, also was suggested to induce premature termination of mRNA synthesis to produce uncapped RNAs with <50 nt (57) (see Supplementary Discussion).

Li *et al*. (34) previously reported that RNA capping with GTP is diminished with the G1154A or T1157A mutation and abolished with the H1227A or R1228A mutation in the VSV L protein. Furthermore, they showed that all these mutant L proteins produce heterogeneous 3′-truncated transcripts (100–500 nt) as well as non-polyadenylated full-length mRNAs in their reconstituted transcription reactions containing rabbit reticulocyte lysates (34,48). However, some of our results are distinctly different from those observations. First, in our hand, the G1154A and T1157A mutants were completely inert in RNA capping with GTP (not shown) as well as GDP (Figure 3), because these mutants were not able to form the L-pRNA intermediate. Second, the G1154A mutant did not synthesize detectable amounts of any transcripts in our reconstituted transcription reaction, although our incubation time (2 h) and amount of the protein (0.15 μg) are shorter and smaller, respectively, than theirs (5 h and 1 or 3 μg). Third, in our transcription reactions, the cap-defective mutants including T1157A, H1227A and R1228A produced uncapped short transcripts with particular lengths (e.g. N1–40, N41–68) and small amounts of 3′-polyadenylated full-length N mRNA (N_1_) and N_2_ RNA, but not heterogeneous 3′-truncated transcripts or non-polyadenylated full-length mRNAs (Figures 4–6) (18). Furthermore, Li *et al*. (34) showed that the G1100A mutant produces capped mRNAs and P1104A is totally inactive in their transcription reactions. However, in our transcription system, the G1100A mutant displayed a typical phenotype of cap-defective mutants producing the uncapped short transcripts and the P1104A mutant is active in transcription although to a lesser extent than the WT L protein. The reasons for these discrepancies are currently not known.

This study shows for the first time that the conserved motifs A, B, C and E as well as motif D in the PRNTase domain of the VSV L protein are essential for the covalent L-pRNA intermediate formation in mRNA capping. Our results also suggest that the successful intermediate formation followed by capping with the PRNTase domain of the L protein licenses the RdRp domain on the same molecule to enter an mRNA chain elongation mode, which enables it to ignore cryptic termination and initiation signals within genes. To understand the molecular basis for co-transcriptional pre-mRNA capping with the highly sophisticated mRNA synthesis machine, further biochemical and structural analyses are necessary. Since all the conserved motifs identified in this study are functionally essential for the PRNTase domain of the VSV L protein, we suggest that this conserved viral enzyme become a potential target for developing broad-spectrum anti-viral agents against significant NNS RNA viruses.

## Supplementary Material

SUPPLEMENTARY DATA
