# Supplementary material for: Signature motifs of GDP polyribonucleotidyltransferase, a non-segmented negative strand RNA viral mRNA capping enzyme, domain in the L protein are required for covalent enzyme–pRNA intermediate formation
Source: Nucleic Acids Res. 2015 Nov 23;44(1):330–41. doi: 10.1093/nar/gkv1286 (PMC4705655; doi:10.1093/nar/gkv1286)
Supplement: SUPPLEMENTARY DATA [file supp_44_1_330__index.html]

Signature motifs of GDP polyribonucleotidyltransferase, a non-segmented negative strand RNA viral mRNA capping enzyme, domain in the L protein are required for covalent enzyme–pRNA intermediate formation — SUPPLEMENTARY DATA 

# Signature motifs of GDP polyribonucleotidyltransferase, a non-segmented negative strand RNA viral mRNA capping enzyme, domain in the L protein are required for covalent enzyme–pRNA intermediate formation

## SUPPLEMENTARY DATA

- SUPPLEMENTARY DATA
- SUPPLEMENTARY DATA
