# Supplementary material for: Signature motifs of GDP polyribonucleotidyltransferase, a non-segmented negative strand RNA viral mRNA capping enzyme, domain in the L protein are required for covalent enzyme–pRNA intermediate formation
Source: Nucleic Acids Res. 2015 Nov 23;44(1):330–41. doi: 10.1093/nar/gkv1286 (PMC4705655; doi:10.1093/nar/gkv1286)
Supplement: SUPPLEMENTARY DATA [file supp_gkv1286_nar-02717-r-2015-File011.pdf]

SUPPLEMENTARY TABLE

Table S1. Conserved amino acid sequences in putative PRNTase domains of NNS RNA viruses and their related viruses

	Abbrev.	Name	Genus	Subfamily	Family	GenBank Acc. No.	Amino acid sequence including motif: A1	A2	B	C	D	E
1	**VSV (VSIV)**	**Vesicular stomatitis Indiana virus**	Vesiculovirus		Rhabdoviridae	K02378	TLRYKSWG	TVIGTTVPHP	PAYLGSKTSE	ISWFV	ALHRFS	NFDFLFQATLLY
2	MORV	Morreton virus	Vesiculovirus*		Rhabdoviridae	KM205007	TLRYKSWG	TVIGTTVPHP	PAYLGSKTSE	ISWFV	ALHRFS	NYDFLFQATLLY
3	MARAV	Maraba virus	Vesiculovirus		Rhabdoviridae	HQ660076	LLRFRSWG	SVIGTTVPHP	PAYLGSKTSE	ISWFV	ALHRFS	NYDFLFQATLLY
4	COCV	Cocal virus	Vesiculovirus		Rhabdoviridae	EU373657	DLRYRSWG	TVIGTTVPHP	PAYLGSKTSE	ISWFI	ALHRFS	NYDFLFQATLLY
5	VSAV	Vesicular stomatitis Alagoas virus	Vesiculovirus		Rhabdoviridae	EU373658	SLRYKSWG	TVIGTTIPHP	PAYLGSRTSE	ISWFV	ALHRFS	NYDFLFQATLLY
6	VSNV	Vesicular stomatitis New Jersey virus	Vesiculovirus		Rhabdoviridae	M29788	ILRYKSWT	KVVGTTVPHP	PAYLGSNTSE	ISWFI	ALHRFS	NYDFMFQASLLY
7	CJSV	Carajas virus	Vesiculovirus		Rhabdoviridae	FW339542	LLRYRSWG	KLVGTTIPHP	PAYLGSKTSE	ISWFI	ALHRFS	NYDFMFQASLLY
8	ISFV	Isfahan virus	Vesiculovirus		Rhabdoviridae	AJ810084	YLRRLSWG	KVLGTTIPHP	AAYLGSKTSE	IHWFV	ALHRFT	NYDFMFQACLLF
9	MSPV	Malpais Spring virus	Vesiculovirus*		Rhabdoviridae	KC412247	LLRKVSWG	PVLGTTIPHP	PAYLGSKTSE	IHWFI	ALHRFT	NFDFMFQASLLY
10	CHPV	Chandipura virus	Vesiculovirus		Rhabdoviridae	AJ810083	KLRRMSWK	PVLGTTVPHP	PAYLGSKTSE	IHWFV	ALHRFS	NYDFMFQASLLY
11	JURV	Jurona virus	Vesiculovirus		Rhabdoviridae	HM566194	SLRRKSWG	QVLGTTIPHP	AAYLGSKTSE	IHWFV	ALHRFS	NFDFMFQASLIY
12	PERV	Perinet virus	Vesiculovirus		Rhabdoviridae	HM566195	LLRKRSWR	RVLGTTVPHP	PAYLGSKTME	IHWFV	ALHRFS	NYDFMFQAGLLY
13	YBV	Yug Bogdanovac virus	Vesiculovirus		Rhabdoviridae	JF911700	YLRTLSWK	KCLGTTVPHP	PAYLGSKTSE	IHWFI	ALHRFS	NYDFMFQASLLY
14	RADIV	Radi virus	Vesiculovirus*		Rhabdoviridae	KM205024	YLRLLSWK	KCLGTTVPHP	PAYLGSKTSE	IHWFV	ALHRFS	NYDFMFQASLLY
15	ABVV	American bat vesiculovirus	Vesiculovirus		Rhabdoviridae	JX569193	YLRVQSWG	PVLGTTVPHP	PAYLGSKTSE	IHWFI	ALHRFT	NFDFMYQASLLY
16	PFRV	Pike fry rhabdovirus	Sprivivirus		Rhabdoviridae	FJ872827	LLREKSWG	SVIGMTVPHP	AAYLGSKTSE	ISWFV	ALHRFT	NYDFMFQAGILY
17	TRV	Tench rhabdovirus			Rhabdoviridae	KC113517	LLREKSWG	SVIGMTVPHP	AAYLGSKTSE	ISWFV	ALHRFT	NYDFMFQAGILY
18	GCRV	Grass carp rhabdovirus			Rhabdoviridae	KC113518	LLREQSWG	SVIGMTVPHP	AAYLGSKTSE	ISWFV	ALHRFT	NYDFMFQAGILY
19	SVCV	Spring viremia of carp virus	Sprivivirus		Rhabdoviridae	AJ318079	LLREKSWG	SVIGMTVPHP	AAYLGSKTSE	ISWFV	ALHRFT	NYDFMFQAGILY
20	EVEX	Eel Virus European X	Perhabdovirus		Rhabdoviridae	FN557213	ELRTLSWG	TIIGTTVPHP	PAYLGSKTSE	ITWFV	ALHRFT	NYDFMFQSLLLF
21	EVA	Eel virus American	Perhabdovirus*		Rhabdoviridae	KC608038	ELRTLSWG	TIIGTTVPHP	PAYLGSKTSE	ITWFV	ALHRFT	NYDFMFQSLLLF
22	PRV	Perch rhabdovirus	Perhabdovirus		Rhabdoviridae	JX679246	ELRQESWG	KVIGTTVPHP	PAYLGSKTSE	ITWFV	ALHRFT	NYDFMFQSLLLY
23	DRV	Dolphin rhabdovirus			Rhabdoviridae	KF958252	LLRFESWG	KVVGATVPHP	PAYLGSKTSE	ISWFI	ALHRFS	NYDFMFQSLLIF
24	SCRV	Siniperca chuatsi rhabdovirus			Rhabdoviridae	DQ399789	MLRQESWG	PVHGATIPHP	PAYLGSKTSE	ISWFV	ALHRFS	NYDFMFQSLLIH
25	SMRV	Scophthalmus maximus rhabdovirus			Rhabdoviridae	HQ003891	RLRSESWG	QVVGTTVPHP	MAYLGSKTSE	ISWFV	ALHRFS	NYDFMFQSLLIH
26	BARV	Barur virus	Ledantevirus*		Rhabdoviridae	KM204983	LLRRISWG	PVLGATVPHP	PAYLGSKTSE	INWFV	ALHRFS	NYDFMFQPSILY
27	FUKV	Fukuoka virus	Ledantevirus*		Rhabdoviridae	KM205001	LLRRISWG	TVLGATVPHP	PAYLGSKTSE	INWFV	ALHRFS	NYDFMFQPSILY
28	NIMV	Nishimuro virus	Ledantevirus*		Rhabdoviridae	AB609604	LLRRISWG	KVLGATVPHP	PAYLGSKTSE	INWFV	ALHRFS	NYDFMFQPSILY
29	NKOV	Nkolbisson virus	Ledantevirus*		Rhabdoviridae	KM205017	LLRRLSWG	EVLGTTIPHP	PAYLGSKTSE	INWFV	ALHRFS	NFDFMFQPTILF
30	KEUV	Keuraliba virus	Ledantevirus*		Rhabdoviridae	KM205021	QLRRMSWG	PVLGTTIPHP	PAYLGSKTSE	IHWFV	ALHRFG	NFDFMFQPSILC
31	LDV	Le Dantec virus	Ledantevirus*		Rhabdoviridae	KM205006	QLRRMSWG	PVLGTTVPHP	PAYLGSKTSE	IHWFV	ALHRFG	NFDFMFQPSILC
32	KCV	Kern Canyon virus	Ledantevirus*		Rhabdoviridae	KM204992	QLRLLSWG	RVLGTTIPHP	PAYLGSKTSE	IHWFV	AIHRFS	NYDFMFQPSILY
33	FKRV	Fikirini bat rhabdovirus	Ledantevirus*		Rhabdoviridae	KC676792	RLRQSSWG	KVVGTTVPHP	TAYLGSRTAE	IHWFV	ALHRFS	NYDFMFQPSILY
34	KOLEV	Kolente virus	Ledantevirus*		Rhabdoviridae	KC984953	RLRYSSWG	KVVGTTIPHP	TAYLGSRTSE	IHWFV	ALHRFS	NYDFMFQPSILY
35	KRV	Kumasi rhabdbovirus			Rhabdoviridae	KJ179955	SLRFRSWG	PVIGTTIPHP	VAYLGSRTSE	IHWFV	ALHRFS	NFDFMFQPSIIY
36	OITAV	Oita virus	Ledantevirus*		Rhabdoviridae	KM204998	NLRLISWG	RVIGTTVPHP	VAYLGSRTSE	IHWFV	ALHRFT	NYDFMFQPSILY
37	MEBV	Mount Elgon bat virus	Ledantevirus*		Rhabdoviridae	KM205026	LLRQISWG	KVIGATVPHP	LAYLGSKTSE	IHWFI	ALHRFS	NYDFMFQSSILF
38	FLAV	Flanders virus	Hapavirus*		Rhabdoviridae	AF523199	ELREKSWG	KVLGTTIPHP	PPYLGSKTSE	ISWFV	ALHRFT	NYDFMFQSLIVY
39	HPV	Hart Park virus	Hapavirus*		Rhabdoviridae	KM205011	QLREESWG	KVLGTTIPHP	PPYLGSKTSE	ISWFV	ALHRFT	NYDFMFQSLIVY
40	KAMV	Kamese virus	Hapavirus*		Rhabdoviridae	KM204989	ELRLKSWN	RVLGTTIPHP	PPYLGSRTSE	ISWFV	ALHRFT	NYDFMFQSLIIY
41	MOSV	Mossuril virus	Hapavirus*		Rhabdoviridae	KM204993	ELRLASWN	KVLGTTIPHP	PPYLGSRTSE	ISWFV	ALHRFT	NYDFMFQSLIIY
42	MQOV	Mosqueiro virus	Hapavirus*		Rhabdoviridae	KM205014	ELRLNSWG	PVLGTTVPHP	PPYLGSRTSE	ISWFI	ALHRFT	NYDFMFQSLIIY
43	LJAV	Landjia virus	Hapavirus*		Rhabdoviridae	KM205010	ELRELSWN	KVLGTTVPHP	PPYLGSKTSE	ISWFV	ALHRFT	NYDFMFQSLIIY
44	MANV	Manitoba virus	Hapavirus*		Rhabdoviridae	KM205008	KLREQSWG	KVLGTTVPHP	PPYLGSKTSE	ISWFV	ALHRFT	NYDFMFQSLIIY
45	GLOV	Gray Lodge virus	Hapavirus*		Rhabdoviridae	KM205022	HLRRISWG	DVLGTTVPHP	HPYLGSRTSE	ISWFV	ALHRFT	NYDFMFQSLIIY
46	ORV	Ord River virus	Hapavirus*		Rhabdoviridae	KM205025	YLRKISWG	DILGMTIPHP	PPYLGSKTSE	ISWFI	ALHRYS	NYDFMFQSLLIY
47	PCV	Parry Creek virus	Hapavirus*		Rhabdoviridae	KM204988	YLRKASWG	DILGMTIPHP	PPYLGSKTSE	ISWFI	ALHRYS	NYDFMFQSLLIY
48	WONV	Wongabel virus	Hapavirus*		Rhabdoviridae	EF612701	YLRRQSWG	DILGMTIPHP	PPYLGSKTSE	ISWFI	ALHRYS	NYDFMFQSLLIY
49	LJV	La Joya virus	Hapavirus*		Rhabdoviridae	KM204986	YLREKSWG	KILGMTIPHP	PPYLGSKTSE	ISWFI	ALHRYS	NFDFMFQSLLIY
50	MCOV	Marco virus	Hapavirus*		Rhabdoviridae	KM205005	DLRKKSWA	PVLGTTIPHP	PPYLGSKTSE	ISWFV	ALHRFT	NYDFMFQSLLIY
51	JOIV	Joinjakaka virus	Hapavirus*		Rhabdoviridae	KM205016	TLREISWG	KVLGTTIPHP	TPYLGSSTSE	ISWFI	AIHRFT	NYDFMFQSLIVL
52	NGAV	Ngaingan virus	Hapavirus*		Rhabdoviridae	FJ715959	ELRLTSWG	KVFGTTVPHP	PPYLGSRTSE	ISWFI	ALHRWS	NYDFMFQSLLIH
53	BEFV	Bovine ephemeral fever virus	Ephemerovirus		Rhabdoviridae	AF234533	SLRKRSWG	EVLGTTVPHP	YPYLGSNTKE	INWFV	SLHRFG	NYDFMFQSTLVW
54	BRMV	Berrimah virus	Ephemerovirus		Rhabdoviridae	HM461974	SLRKRSWG	EVLGTTVPHP	YPYLGSNTKE	INWFV	SLHRFG	NYDFMFQSTLVW
55	KIMV	Kimberley virus	Ephemerovirus*		Rhabdoviridae	JQ941664	KLRFRSWG	EVLGTTVPHP	YPYLGSNTKE	INWFV	SLHRFS	NYDFMFQSTIIW
56	MALV	Malakal virus	Ephemerovirus*		Rhabdoviridae	JQ941707	KLRFRSWG	EVLGTTVPHP	YPYLGSNTKE	INWFV	SLHRFS	NYDFMFQSTIIW
57	KOTV	Kotonkan virus	Ephemerovirus		Rhabdoviridae	HM474855	HLRLLSWG	KVLGMTVPHP	NPYLGSKTKE	ISWFI	ALHRFS	NYDFMYQSSMIY
58	KOOLV	Koolpinyah virus	Ephemerovirus*		Rhabdoviridae	KM085029	HLRTLSWG	KVLGMTIPHP	SPYLGSKTKE	ISWFI	ALHRFS	NYDFMYQSSMIY
59	YATV	Yata virus	Ephemerovirus*		Rhabdoviridae	KM085030	YLRRLSWN	SVLGMTIPHP	SPYLGSRTLE	ISWFI	PLHRFT	NYDFMYQASMIY
60	OBOV	Obodhiang virus	Ephemerovirus		Rhabdoviridae	HM856902	RLRKLSWN	PIVGATIPHP	DPYLGSRTRE	INWFI	ALHRFG	NYDFMHQTLIIY

Table S1. (continued)

	Abbrev.	Name	Genus	Subfamily	Family	GenBank	Amino acid sequence including motif:					
			(*: proposed)			Acc. No.	A1	A2	B	C	D	E
61	ARV	Adelaide River	Ephemerovirus		Rhabdoviridae	JN935380	RLRRLSWN	VIVGATIPHP	DPYLGSRTRE	INWFI	ALHRFG	NYDFMYQTLIIF
62	TIBV	Tibrogargan virus	Tibrovirus		Rhabdoviridae	GQ294472	KLRKQSWN	DIIGMTVPHP	KPYLGSSTNE	ISWFV	ALHRFR	NYDFMFQASIIF
63	BAV	Bivens Arm virus	Tibrovirus*		Rhabdoviridae	KP688373	RLRKQSWN	DIIGMTVPHP	KPYLGSSTNE	ISWFV	ALHRFR	NYDFMFQASIIF
64	SWBV	Sweetwater Branch virus	Tibrovirus*		Rhabdoviridae	KM204997	ELRKRSWG	DIVGMTVPHP	KPYLGSSTNE	ISWFV	ALHRFR	NYDFMFQASIIY
65	CPV	Coastal Plains virus	Tibrovirus		Rhabdoviridae	GQ294473	RLRQMSWG	RIIGITVPHP	RPYLGSGTSE	ISWFV	ALHRFK	NYDFMFQASMIF
66	EKV-1	Ekpoma virus-1	Tibrovirus*		Rhabdoviridae	KP324827	MLRRKSWN	EIIGTTVPHP	PPYTGSSTSE	ISWFV	PLHRFK	NFDFMFQPLMLY
67	TUPV	Tupaia rhabdovirus	Tupavirus		Rhabdoviridae	AY840978	RLRRLSWG	PVIGATIPHP	LPYLGSKTSE	INWFV	ALHRFA	NYDFMFQSLILY
68	KLAV	Klamath virus	Tupavirus*		Rhabdoviridae	KM204999	QLRRESWG	RVIGATIPHP	LPYLGSRTSE	INWFV	AIHRFS	NYDFMFQSLILF
69	DURV	Durham virus	Tupavirus		Rhabdoviridae	FJ952155	ALRMISWG	KVIGSTVPHP	LPYLGSATKE	INWFV	ALHRFG	NYDFMFQSSILF
70	XIBV	Xiburema virus			Rhabdoviridae	KJ636781	LLRRRSWG	NIVGMTVPHP	VPYLGSNTSE	ISWFV	AIHRFS	NYDFMFQSMLIW
71	ARUV	Aruac virus			Rhabdoviridae	KM204987	LLRNNSWG	NIVGMTVPHP	VPYLGSNTSE	ISWFI	AIHRFS	NYDFMFQSMLIW
72	INHV	Inhangapi virus			Rhabdoviridae	KM204991	RLRRESWG	EIVGMTVPHP	VPYLGSNTSE	ISWFV	AIHRFS	NYDFMFQSLLLW
73	SBV	Santa barbara virus			Rhabdoviridae	KM350503	QLRKRSWG	EIVGMTVPHP	DPYLGSNTSE	ISWFV	AIHRFS	NYDFMFQSLILW
74	DObsSV	Drosophila obscura sigma virus	Sigmavirus		Rhabdoviridae	GQ410979	KLRRISWG	EIYGATIPHP	KAYLGSKTSE	IGWFI	ALHRYG	NHNIMFQTFLIY
75	DAffSV	Drosophila affinis sigma virus	Sigmavirus		Rhabdoviridae	GQ410980	KLRKLSWG	KVIGATIPHP	TAYLGSSTSE	IGWFV	GLHRYI	NYDFMFQALIIY
76	BASV	Bas-Congo virus	Tibrovirus*		Rhabdoviridae	JX297815	KLREISWR	RVYGMTVPHP	PPYLGSKTKE	ISWFV	ALHRFQ	NYDFMYQPSMIY
77	EKV-2	Ekpoma virus-2	Tibrovirus*		Rhabdoviridae	KP324828	QLRECSWG	PVLGMTIPHP	PPYLGSNTKE	ICWFV	ALHRFH	NYDFMFQSSLIF
78	DMelSV	Drosophila melanogaster sigma virus	Sigmavirus		Rhabdoviridae	GQ375258	YLRRVSWQ	VVYGATIPHP	VAYLGSSTTE	IGWFI	ALHRFS	NYDFMYQSCLLN
79	CURV	Curionopolis virus	Curiovirus*		Rhabdoviridae	KJ701190	VLRKRSWG	DIVGMTIPHP	EPYLGSNTSE	ISWFI	ALHRFG	NHDFMYQSLFIH
80	RBUV	Rochambeau virus	Curiovirus*		Rhabdoviridae	KM205012	DLRRRSWG	EIVGMTIPHP	EPYLGSNTSE	ISWFV	ALHRFG	NHDFMYQSLFIY
81	IRIRV	Iriri virus	Curiovirus*		Rhabdoviridae	KM204995	ELRRRSWG	EVIGMTIPHP	EPYLGSTTSE	ISWFV	ALHRFG	NYDFMYQSLFIY
82	ITAV	Itacaiunas virus	Curiovirus*		Rhabdoviridae	KM204984	DLRKRSWG	TVTGMTVPHP	SPYLGSHTSE	ISWFV	AHHRFS	NLDFMYQSLFVY
83	LSRV-No9	Lepeophtheirus salmonis rhabdovirus No9			Rhabdoviridae	KJ958535	LLRKLSWG	DLVGTTVPHP	QPYLGSKTSE	ISWFV	ALHRFQ	NYDFMFQSLLLY
84	LSRV-No127	Lepeophtheirus salmonis rhabdovirus No127			Rhabdoviridae	KJ958536	YLRESSWA	PLVGTTVPHP	SPYLGSKTSE	ISWFV	ALHRFH	NYDFIFQSLILY
85	NORCV	North Creek virus			Rhabdoviridae	KF360973	SLRTRSWN	AVIGTTVPHP	PSYLGSSTGE	FHWFV	SLHRFG	NYDFMYQSLMLY
86	HARDV	Harrison Dam virus			Rhabdoviridae	KJ432573	MLRKISWG	DIVGMTIPHP	IPYLGSATSE	INWFV	AIHRFS	NWDFMFQSLIIH
87	WACV	Walkabout Creek virus			Rhabdoviridae	KJ432572	VLRKISWS	NIVGMTIPHP	IPYLGSTTSE	INWFV	AIHRFS	NWDFMFQSLIIH
88	SUNV	Sunguru virus			Rhabdoviridae	KF395226	LLRKLSWG	EIVGMTIPHP	TPYLGSKTSE	INWFV	AIHRFS	NWDFMFQSLIIM
89	GARV	Garba virus			Rhabdoviridae	KM204982	QLREISWG	EIVGMTIPHP	IPYLGSQTSE	LNWFI	AIHRFS	NYDFMYQSAIIS
90	OVRV	Oak-Vale virus			Rhabdoviridae	JF705877	SLRKRSWG	EIVGVTIPHP	VPYLGSATSE	IGWFV	AIHRYG	NYDFMFQANIIF
91	KWAV	Kwatta virus			Rhabdoviridae	KM204985	SLRKRSWG	DIVGVTIPHP	VPYLGSATSE	LGWFV	AIHRYG	NYDFMFQANIIF
92	EBLV2	European bat lyssavirus 2	Lyssavirus		Rhabdoviridae	EF157977	LLREISWG	KVVGTTVPHP	KGYLGSSTSM	INWFI	ALHRFK	NFDFMFQPLMLY
93	KHUV	Khujand lyssavirus	Lyssavirus		Rhabdoviridae	EF614261	LLREISWG	KVVGTTVPHP	KGYLGSSTSM	INWFI	ALHRFK	NFDFMFQPLMLY
94	BBLV	Bokeloh bat lyssavirus	Lyssavirus		Rhabdoviridae	JF311903	LLREISWG	KVVGTTVPHP	KGYLGSSTSM	INWFI	ALHRFK	NFDFMFQPLMLY
95	ARAV	Aravan virus	Lyssavirus		Rhabdoviridae	EF614259	QLREISWG	KVVGTTVPHP	KGYLGSSTSM	INWFI	ALHRFK	NFDFMFQPLMLY
96	ABLV	Australian bat lyssavirus	Lyssavirus		Rhabdoviridae	AF418014	LLREISWG	KVVGTTVPHP	KGYLGSSTSM	INWFI	ALHRFK	NFDFMFQPLMLY
97	IRKV	Irkut virus	Lyssavirus		Rhabdoviridae	EF614260	QLREISWG	KVVGTTVPHP	KGYLGSSTSM	INWFI	ALHRFK	NFDFMFQPLMLY
98	OZEV	Ozernoe virus	Lyssavirus		Rhabdoviridae	FJ905105	QLREISWG	KVVGTTVPHP	KGYLGSSTSM	INWFI	ALHRFK	NFDFMFQPLMLY
99	EBLV1	European bat lyssavirus 1	Lyssavirus		Rhabdoviridae	EF157976	QLREISWG	KVVGTTVPHP	KGYLGSSTSM	INWFI	ALHRFK	NFDFMFQPLMLY
100	DUVV	Duvenhage virus	Lyssavirus		Rhabdoviridae	EU293120	QLREISWG	KVVGTTVPHP	KGYLGSSTSM	INWFI	ALHRFK	NFDFMFQPLMLY
101	RABV	Rabies virus	Lyssavirus		Rhabdoviridae	M13215	LLREISWG	KVVGTTVPHP	KGYLGSSTSM	INWFI	ALHRFK	NYDFMFQPLMLY
102	LBV	Lagos bat virus	Lyssavirus		Rhabdoviridae	EU293110	QLREISWG	KVVGTTVPHP	KGYLGSSTSV	INWFV	ALHRFK	NFDFMFQPLMLY
103	SBV	Shimoni bat virus	Lyssavirus		Rhabdoviridae	GU170201	QLREISWG	KVVGTTVPHP	RGYLGSSTSV	INWFI	ALHRFK	NFDFMFQPLMLY
104	MOKV	Mokola virus	Lyssavirus		Rhabdoviridae	Y09762	QLREISWG	KVVGTTVPNP	KGYLGSSTSM	INWFV	ALHRFK	NFDFMFQPLMLY
105	WCBV	West Caucasian bat virus	Lyssavirus		Rhabdoviridae	EF614258	QLREISWG	KVVGTTVPHP	KGYLGSSTSV	INWFI	ALHRFK	NFDFMFQPLMLY
106	IKOV	Ikoma lyssavirus	Lyssavirus		Rhabdoviridae	JX193798	RLREISWG	KVVGTTVPHP	KGYLGSSTSV	INWFI	ALHRFK	NFDFMFQPLMLY
107	CNTV	Connecticut virus	Sawgravirus*		Rhabdoviridae	KM205020	TLRALSWG	KVEGSTVPHP	MPYLGSKTSE	IDWLM	GRHRLR	NHDFMYQSVICW
108	LITRV	Long Island tick rhabdovirus			Rhabdoviridae	KJ396935	NLRCSSWG	KVEGSTIPHP	MPYLGSKTSE	IDWLM	GRHRLR	NHDFMYQSVICW
109	SAWV	Sawgrass virus	Sawgravirus*		Rhabdoviridae	KM205013	LLRSLSWG	KIEGSTVPHP	MPYLGSKTSE	IDWLM	GRHRLR	NHDFMYQSVICW
110	NMV	New Minto virus	Sawgravirus*		Rhabdoviridae	KM205009	QLRLLSWG	TIEGSTIPHP	TPYLGSKTSE	IDWLM	GKHRLR	NHDFMYQSLICW
111	MOUV	Moussa virus	Sawgravirus*		Rhabdoviridae	FJ985748	QLRKQSWG	PLIGATIPHP	MPYLGSKTSE	IDWIT	GQHRLR	NYDFMYQSLLCW
112	BGV	Bahia Grande virus	Bahiavirus*		Rhabdoviridae	KM205018	EMRKLSWG	DMVGITTPHP	MPYLGTKTKE	IRWFV	SKHRLR	NYDFMYQSILCW
113	HALV	Harlingen virus	Bahiavirus*		Rhabdoviridae	KM205003	EMRKLSWG	DMVGITTPHP	MPYLGTKTKE	IRWFV	SKHRLR	NYDFMYQSILCW
114	MSV	Muir Springs virus	Bahiavirus*		Rhabdoviridae	KM204990	EMRKLSWG	KMVGITTPHP	MPYLGTKTKE	IRWFV	SKHRLR	NYDFMYQSILCW
115	CTRV	Culex tritaeniorhynchus rhabdovirus			Rhabdoviridae	AB604791	ELRETSWG	PVVGATIPHP	NPYRGSMTAE	ITWVV	GIHRIR	NYDFMYQALLIF
116	NIAV	Niakha virus	Sripuvirus*		Rhabdoviridae	KC585008	RLRLESWG	KIYGITVTHP	PSYLGSKTAE	MGWFV	AAHRYG	NYDVMFQSVILY
117	SRIV	Sripur virus	Sripuvirus*		Rhabdoviridae	KM205023	HLRMTGWG	KIYGVTVTHP	PSYLGSKTSE	LGWFV	AAHRYG	NYDVMFQSVILY
118	ALMV	Almpiwar virus			Rhabdoviridae	KJ399977	ELRSMSWG	KIYGITIPHP	RSYLGSRTRE	IGWFV	AQHRYG	NYDFMFQATILY
119	CHOV	Chaco virus	Sripuvirus*		Rhabdoviridae	KM205000	QLRREGWG	KIYGVTVVHP	PSYLGSKTAE	IGWFT	SQHRYG	NCDFMYQASILF
120	SMV	Sena Madureira virus	Sripuvirus*		Rhabdoviridae	KM205004	QLRREGWG	IIYGVTVVHP	PSYLGSKTSE	IGWFT	SQHRYG	NCDFMYQASMLY

Table S1. (continued)

	Abbrev.	Name	Genus (*: proposed)	Subfamily	Family	GenBank Acc. No.	Amino acid sequence including motif: A1	A2	B	C	D	E
121	ABTV	Arboretum virus	Almendravirus*		Rhabdoviridae	KC994644	YLRQWTWE	EIVGSTIPHP	KPYLGSNTKE	INWLT	AEHRYR	NYDFMFQASMIY
122	PTAMV	Puerto Almendras virus	Almendravirus*		Rhabdoviridae	KF534749	YLRRTTWG	EIIGSTIPHP	RPYLGSNTKE	INWLT	AEHRYR	NYDFMYQASLIY
123	RFFRV	Red fox fecal rhabdovirus			Rhabdoviridae	KF823814	SYRSKSWG	QIWGVTVPHP	NPYLGGRTSE	CGWAY	LGHRFS	NYMVLFQGLFVY
124	MMV	Maize mosaic virus	Nucleorhabdovirus		Rhabdoviridae	AY618418	RYRYYSWK	KVLGVTVPHP	KIYQGSYTKE	INWRY	FDHRRG	NENIHFQSVLIN
125	TaVCV	Taro vein chlorosis virus	Nucleorhabdovirus		Rhabdoviridae	AY674964	QYRDLSWR	PILGVTVPHP	KIYQGSYTKE	INWRY	FDHRRG	NENIHFQSVLIN
126	IMMV	Iranian maize mosaic nucleorhabdovirus	Nucleorhabdovirus		Rhabdoviridae	DQ186554	RYRLYSWR	PVIGVTVPHP	KIYQGSYTKE	INWRY	FDHRRG	NENIHFQSVLIN
127	PYDV	Potato yellow dwarf virus	Nucleorhabdovirus		Rhabdoviridae	GU734660	VARQMSYG	RILGTTVPHP	KPYYGSYTKE	IGWRY	YDHRRN	NEYIHFQACIIT
128	EMDV	Eggplant mottled dwarf virus	Nucleorhabdovirus		Rhabdoviridae	KC905081	SMRSLSYK	QVLGVTVPHP	KPYFGSYTKE	IGWRY	YDHRRN	NEYIHFQSCMIT
129	RYSV	Rice yellow stunt virus	Nucleorhabdovirus		Rhabdoviridae	AB011257	ECRTKGWG	PVLGVTVPTP	KPYLGAYTKE	INWRY	YDHRRK	NENIHFQACIIQ
130	OFV	Orchid fleck virus‡	Dichorhavirus*			AB244418	RYRRLSWG	EILSITTPHP	PVYMGSYTRE	INWRY	AEHRYQ	NETLHFQAVMIY
131	CoRSV	Coffee ringspot virus‡	Dichorhavirus*			KF812526	RYRAISWG	NIMGITTPHP	HVYQGSYTKE	LSRRY	AEHRYQ	NETIHFQAVLIY
132	SYNV	Sonchus yellow net virus	Nucleorhabdovirus		Rhabdoviridae	L32603	MLRNWTWG	NIIGVTTPHP	RPYFGSYTEE	LGWRY	VEHRYH	NESLHFQAAIIW
133	MFSV	Maize fine streak virus	Nucleorhabdovirus		Rhabdoviridae	AY618417	IYRSTSWK	PIVGVTVPSP	EPYLGSFTDE	RYWRY	ATHRYS	NDMIHFQGSLLW
134	LNYV	Lettuce necrotic yellows virus	Cytorhabdovirus		Rhabdoviridae	AJ867584	QVRRIGWG	ELIGVTVAYP	PPYLGSMTKE	IGWFV	EIHRFR	NYDMLFQANLCA
135	LYMoV	Lettuce yellow mottle virus	Cytorhabdovirus		Rhabdoviridae	EF687738	QMRYLGWG	ELIGVTVAFP	PPYLGSVTKE	IGWFV	EIHRFR	NYDMLFQANLCA
136	PeVA	Persimmon virus A	Cytorhabdovirus*		Rhabdoviridae	AB735628	KMREKSWG	NLRGITTPFP	PPYLGSMTKE	INWYV	EVHRYR	NYDVHFQACLCS
137	ADV	Alfalfa dwarf virus	Cytorhabdovirus*		Rhabdoviridae	KP205452	EMRQEGWK	NLRGITIPHP	PPYLGSMTKE	INWFV	EVHRYK	NTDLHFQALYCY
138	NCMV	Northern cereal mosaic virus	Cytorhabdovirus		Rhabdoviridae	AB030277	HLRKKGWK	DVVGISTPYP	LPYLGSVTKE	IGWFI	MMHRYL	NVTLQFQAMLGL
139	BYSMV	Barley yellow striate mosaic virus	Cytorhabdovirus		Rhabdoviridae	KM213865	YLRDKGWK	KVIGISTPYP	LPYLGSVTKE	IGWFI	MMHRYV	NVTLHFQALLGL
140	FARV	Farmington virus	Cytorhabdovirus*		Rhabdoviridae	KC602379	QYRNQSWG	QIHGVSVGHP	YPYIGSETRE	INWFI	AVHRYS	NTDAHFQSMMIY
141	ScRV	Soybean cyst nematode associated northern cereal mosaic virus	Cytorhabdovirus*		Rhabdoviridae	HM849039	YLRTMGWQ	QIEGVTTPLP	SGYFGGETRQ	LGWLA	AGHRLQ	DFPIHYQQLICY
142	Sf-RV	Spodoptera frugiperda rhabdovirus			Rhabdoviridae	KF947078	FLRNMGWG	IIEGVDSAPP	RPYRGSITKN	ESWAF	YQHRLR	NKNVMFQGPLVM
143	PoRV	Porcine rubulavirus	Rubulavirus	Paramyxovirinae	Paramyxoviridae	BK005918	TLRKLSWA	PIEGLETPDP	VPYIGSKTDE	YIWAF	ITHRLD	DCNLIYQQIMLL
144	MPRV	Mapuera virus	Rubulavirus	Paramyxovirinae	Paramyxoviridae	EF095490	TLRKLSWA	PIEGLETPDP	VPYIGSRTDE	YIWAF	ITHRLD	DSNLIYQQLMIL
145	MuV	Mumps virus	Rubulavirus	Paramyxovirinae	Paramyxoviridae	D10575	SLRKLSWA	PIEGLETPDP	VPYIGSKTDE	YIWAF	LVHRLD	DSNIIYQQLMLL
146	BatPV_Epo	Bat Paramyxovirus Epo		Paramyxovirinae	Paramyxoviridae	HQ660095	SLRKLSWA	PIEGLETPDP	VPYIGSKTDE	YIWAF	LVHRLD	DSNLIYQQVMIL
147	HPIV2	Human parainfluenza virus 2	Rubulavirus	Paramyxovirinae	Paramyxoviridae	X57559	SLRKLSWS	TLEGLETPDP	VPYIGSRTEE	YIWAF	ITHRLD	DSNLIYQQVMIL
148	SV41	Simian virus 41	Rubulavirus	Paramyxovirinae	Paramyxoviridae	X64275	SLRKLSWS	TLEGLETPDP	VPYVGSKTEE	YIWAY	ITHRLD	DSNLIYQQVMLL
149	PIV5	Parainfluenza virus 5/simian virus 5	Rubulavirus	Paramyxovirinae	Paramyxoviridae	AF052755	NLRKLSWA	NLEGLETPDP	VPYIGSRTDE	YIWAF	LTHRLD	DSNLIYQQIMLL
150	HPIV4a	Human parainfluenza virus 4a	Rubulavirus	Paramyxovirinae	Paramyxoviridae	AB543336	NLRKLSWA	GLEGLETPDP	VPYIGSKTDE	YIWAF	LTHRLD	DSNLIYQQIMLL
151	ThkPV-2	Tuhoko virus 2	Rubulavirus*	Paramyxovirinae	Paramyxoviridae	GU128081	LLRRMSWS	PIDGLETPDP	VPYIGSRTDE	YIWAF	LTHRLD	DSNLIYQQIMLL
152	AchPV1	Achimota virus 1	Rubulavirus*	Paramyxovirinae	Paramyxoviridae	JX051319	LLRKLSWS	GIDGLETPDP	VPYIGSRTDE	YIWAF	LTHRLD	DSNLIYQQVMIT
153	AchPV2	Achimota virus 2	Rubulavirus*	Paramyxovirinae	Paramyxoviridae	JX051320	VVRRLSWL	SIDGLETPDP	VPYVGSKTDE	YIWAF	LTHRLD	DSNLIYQQVMIT
154	ThkPV-3	Tuhoko virus 3	Rubulavirus*	Paramyxovirinae	Paramyxoviridae	GU128082	LLRRLSWS	PLEGLETPDP	VPYVGSRTDE	YIWAF	LTHRLD	DSNLIYQQVMIT
155	SosV	Sosuga virus	Rubulavirus*	Paramyxovirinae	Paramyxoviridae	KF774436	ILRRISWS	PLEGLETPDP	VPYVGSRTDE	YIWAF	LTHRLD	DSNLIYQQVMIT
156	ThkPV-1	Tuhoko virus 1	Rubulavirus*	Paramyxovirinae	Paramyxoviridae	GU128080	LLRKLSWT	GLQGLETPDP	VPYVGSRTDE	YIWAF	LVHRLD	DSNIIYQQLMIT
157	TiV	Tioman virus	Rubulavirus*	Paramyxovirinae	Paramyxoviridae	AF298895	LVRRLSWK	QLDGLETPDP	VPYIGSRTDE	FIWAY	LTHRLD	DSNIIYQQLMIT
158	TevPV	Teviot virus			Paramyxoviridae	KP271123	LARRLSWR	PLDGLETPDP	VPYIGSRTDE	YIWAF	LTHRLD	DSNIIYQQLMIT
159	MenV	Menangle virus	Rubulavirus*	Paramyxovirinae	Paramyxoviridae	AF326114	MVRRLSWS	QLDGLETPDP	VPYIGSRTDE	YIWAF	LTHRLD	DSNLIYQQIMIT
160	APMV2	Avian paramyxovirus 2	Avulavirus	Paramyxovirinae	Paramyxoviridae	EU338414	FLRATSWS	TISGVTSPDT	VPYLGSKTEE	MVWAF	IQHRLN	DSNLIYQQIMLT
161	APMV10	Avian paramyxovirus 10	Avulavirus*	Paramyxovirinae	Paramyxoviridae	HM147142	FLRASSWK	GVAGVTSPDT	VPYLGSKTEE	YMWAF	IQHRLT	DSNLIYQQIMLI
162	APMV8	Avian paramyxovirus 8	Avulavirus	Paramyxovirinae	Paramyxoviridae	FJ215863	YLRAVSWS	NISGVTTPDT	VPYLGSKTEE	FIWAF	IQHRLD	DSNLIYQQIMLL
163	APMV11	Avian paramyxovirus 11	Avulavirus*	Paramyxovirinae	Paramyxoviridae	JQ886184	FTRATSWS	RISGITTPDT	VPYVGSKTEE	YIWAY	IQHRLI	ESNLIYQQIMLL
164	APMV5	Avian paramyxovirus 5	Avulavirus	Paramyxovirinae	Paramyxoviridae	GU206351	YTRSNSWR	QIIGVTSPDT	IAYVGSKTEE	YIWAF	IQHRLE	ESNIIYQQVMIL
165	APMV6	Avian paramyxovirus 6	Avulavirus	Paramyxovirinae	Paramyxoviridae	AY029299	YARTYSWW	SIKGVTTPDT	IAYVGSKTEE	YIWAY	IQHRLM	ESNVIFQQVMLL
166	APMV7	Avian paramyxovirus 7	Avulavirus	Paramyxovirinae	Paramyxoviridae	FJ231524	FTRTCSWS	QIVGVTTPDT	VPYLGSKTDE	YVWAY	LQHRLA	ESNLIYQQIMLL
167	NDV	Newcastle disease virus	Avulavirus	Paramyxovirinae	Paramyxoviridae	AY262106	YARNRSWS	KILGVSNPDT	VPYLGSKTQE	LIWAY	LQHRLD	EGNVVYQQIMLL
168	PPMV1	Pigeon paramyxovirus-1	Avulavirus	Paramyxovirinae	Paramyxoviridae	GQ429292	YARNRSWS	KILGVSNPDT	VPYLGSKTQE	LIWAY	LQHRLD	EGNVVYQQIMLL
169	APMV9	Avian paramyxovirus 9	Avulavirus	Paramyxovirinae	Paramyxoviridae	EU910942	YLRQITWL	KILGVNNPDT	IPYVGSKTQE	KVRAY	LQHRLD	ESNIIYQQIMLL
170	APMV12	Avian paramyxovirus 12	Avulavirus*	Paramyxovirinae	Paramyxoviridae	KC333050	YCRNRSWS	PILGVSNPDS	VPYLGSKTQE	LIWAF	LQHRLD	ESNLIYQQIMLL
171	APMV4	Avian paramyxovirus 3	Avulavirus	Paramyxovirinae	Paramyxoviridae	EU403085	QVRRASWA	SLEGLEVPDP	VPYIGSKTDE	YIWAF	LHHRLN	DSNLIYQQVMLL
172	APMV3	Avian paramyxovirus 4	Avulavirus	Paramyxovirinae	Paramyxoviridae	EU877976	VSRRISWA	AIDGLETPDV	MPYVGSKTDE	YLWAY	LHHRLN	DSNMIYQQVMIL
173	RPV	Rinderpest virus	Morbillivirus	Paramyxovirinae	Paramyxoviridae	X98291	ALRSHMWA	PIYGLEVPDV	VPYIGSTTDE	YSWAY	LAHRLR	DTNFIYQQGMLL
174	MeV	Measles virus	Morbillivirus	Paramyxovirinae	Paramyxoviridae	M20865	ALRSHMWA	PIYGLEVPDV	VPYIGSTTDE	YSWAY	LAHRLR	DTNFIYQQGMLL
175	PPRV	Peste des petits ruminants virus	Morbillivirus	Paramyxovirinae	Paramyxoviridae	AJ849636	ALRSHMWA	PIYGLEVPDI	VPYVGSTTEE	YSWAY	LAHRLR	DTNFIYQQGMLL
176	PDV	Phocine distemper virus	Morbillivirus	Paramyxovirinae	Paramyxoviridae	Y09630	ALRNHMWA	PIYGLEVPDI	VPYIGSTTEE	YSWAY	LAHRLR	DTNFIYQQGMLL
177	CDV	Canine distemper virus	Morbillivirus	Paramyxovirinae	Paramyxoviridae	Y09629	ALRNHMWA	PIYGLEVPDI	VPYIGSTTEE	YSWAY	LAHRLR	DTNFIYQQGMLL
178	DMV	Dolphin morbillivirus	Morbillivirus	Paramyxovirinae	Paramyxoviridae	AJ608288	ALRSHMWA	SIYGLEVPVV	VPYIGSTTEE	YSWAY	LAHRLR	DTNFIYQQGMLL
179	FmoPV	Feline morbillivirus	Morbillivirus	Paramyxovirinae	Paramyxoviridae	JQ411014	TIRKRMWV	EIYGLEVPDI	VPYVGSTTEE	YTWAY	LAHRLR	DTNFVYQQGMLL
180	TlmPV	Tailam virus		Paramyxovirinae	Paramyxoviridae	JN689227	RLRNVMWN	PIYGLEVPDS	VPYFGSTTEE	YTWAY	IAHRLR	DTNLIYQQVMLL

Table S1. (continued)

	Abbrev.	Name	Genus (*: proposed)	Subfamily	Family	GenBank Acc. No.	A1	A2	B	C	D	E
181	BeV	Beilong virus		Paramyxovirinae	Paramyxoviridae	DQ100461	RIRNVMWN	PIYGLEVPDT	VPYFGSTTEE	YTWAY	IAHRLR	DTNLVYQQVMLL
182	JV	J-virus		Paramyxovirinae	Paramyxoviridae	AY900001	RLRNTMWV	PIYGLEVPDT	VPYFGSTTEE	YTWAY	IAHRLR	DTNLIYQQVMLL
183	NarPV	Nariva virus			Paramyxoviridae	FJ362497	AMRQHMWQ	LIYGLEVPDI	VPYIGSTTEE	YTWAY	LAHRMR	DTNYVYQQGMLL
184	MoV	Mossman virus		Paramyxovirinae	Paramyxoviridae	AY286409	ALRQHMWG	PIYGLEVPDI	VPYVGSSTDE	YTWAF	LAHRMR	DTNYVYQQGMLL
185	TPMV	Tupaia paramyxovirus		Paramyxovirinae	Paramyxoviridae	AF079780	AFRQHMWI	VIYGLETPDV	VPYVGSTTEE	YTWAY	IAHRLR	DTNFVYQQFMLT
186	HeV	Hendra virus	Henipavirus	Paramyxovirinae	Paramyxoviridae	AF017149	ALRCHMWR	SIYGLEVPDA	VPYVGSSTDE	YTWAY	LSHRLR	DTNLIYQQTMLL
187	NiV	Nipah virus	Henipavirus	Paramyxovirinae	Paramyxoviridae	AF212302	ALRSHMWR	VIYGLEVPDA	VPYVGSSTDE	YTWAY	LSHRLR	DTNLIYQQAMLL
188	CedPV	Cedar virus	Henipavirus	Paramyxovirinae	Paramyxoviridae	JQ001776	ALRSRMWR	LIYGLEVPDP	VPYVGSTTEE	YTWAF	IAHRLR	DTNLIYQQVMLL
189	BatPV_Eid	Bat Paramyxovirus Eid			Paramyxoviridae	HQ660129	ALRTHMWR	EIYGLEVPDP	VPYIGSTTEE	YTWAF	LFHRLR	DTNFIFQQSMLL
190	MojV	Mojiang paramyxovirus	Henipavirus*	Paramyxovirinae	Paramyxoviridae	KF278639	YLRSHMWR	LIYGLEVPDP	VPYIGSSTDE	YTWAF	LAHRLR	DTNLIFQQVMLL
191	SalPV	Salem virus		Paramyxovirinae	Paramyxoviridae	JQ697837	MLRHQMRR	PIYGLEVPDM	VPYTGSVTEE	YTWAF	LAHRLR	DTNFIYQQSMLL
192	FDLV	Fer-de-Lance virus	Ferlavirus	Paramyxovirinae	Paramyxoviridae	AY141760	IMRSRMWV	RIEGLEVPDP	VPYFGSSTSE	YTWAY	LSHRLN	DTNLIYQQVMLV
193	AnaPV	Anaconda paramyxovirus	Ferlavirus	Paramyxovirinae	Paramyxoviridae	KJ956404	IMRSRMWV	RIEGLEVPDP	VPYFGSSTSE	YTWAY	LSHRLN	DTNLIYQQVMLV
194	HPIV1	Human parainfluenza virus 1	Respirovirus	Paramyxovirinae	Paramyxoviridae	AF117818	GLRQKMWF	PIHGLETPDP	IPYFGSATDE	YTWAY	LSHRLR	DTNLVYQQIMLT
195	SeV	Sendai virus	Respirovirus	Paramyxovirinae	Paramyxoviridae	X03614	GLRQKMWI	PIHGLETPDP	IPYFGSATDE	YTWAY	LSHRLK	DTNLVYQQIMLT
196	PPIV1	Porcine parainfluenza virus 1	Respirovirus	Paramyxovirinae	Paramyxoviridae	JX857409	GLRQKMWS	PIHGLETPDP	IPYFGSTTDE	YTWAY	LSHRLK	DTNLIYQQIMLT
197	HPIV3	Human parainfluenza virus 3	Respirovirus	Paramyxovirinae	Paramyxoviridae	M21649	ALRQKMWI	MISGLETPDP	VPYFGSVTDE	YTWAF	LSHRLK	DTNLIYQQIMLT
198	BPIV3	Bovine parainfluenza virus 3	Respirovirus	Paramyxovirinae	Paramyxoviridae	D84095	SLRQKMWM	LINGLETPDP	VPYFGSVTDE	YTWAF	LSHRLK	DTNLIYQQVMLT
199	ASPV	Atlantic salmon paramyxovirus	Aquaparamyxovirus	Paramyxovirinae	Paramyxoviridae	EF646380	IIRTKLWC	HIAGLETPDP	VPYFGSTTEE	YTWAF	LSHRLR	DTNLVYQQVMLI
200	SSV	Sunshine virus			Paramyxoviridae	JN192445	KLRSMRGK	MCKGKENLVF	SPYLGSSTEE	SKWIT	FDHRLN	DTNVIYQNCIIH
201	BEVOV	Bundibugyo ebolavirus	Ebolavirus		Filoviridae	FJ217161	ILREYTWA	QLIGATLPCM	VPYIGSRTED	LTWVT	IVHRYN	DSNIIFQNVINF
202	TAFV	Tai Forest ebolavirus/Cote d'Ivoire ebolavirus	Ebolavirus		Filoviridae	FJ217162	ILREYTWA	QLIGATLPCI	IPYIGSRTED	LTWVT	IVHRYN	DSNIIFQNVINF
203	ZEBOV	Zaire ebolavirus	Ebolavirus		Filoviridae	AF086833	ILREYSWA	PLIGATLPCM	IPYIGSRTED	LTWVT	IVHRYN	DSNIIFQNVINY
204	SEBOV	Sudan ebolavirus	Ebolavirus		Filoviridae	U23458	ILREYSWA	QLIGATLPCI	IPYIGSRTED	LTWVT	IVHRYN	DSNIIFQNVINF
205	REBOV	Reston ebolavirus	Ebolavirus		Filoviridae	AF522874	ILREYTWS	SLIGATLPCM	VPYIGSRTED	LTWVT	IVHRYN	DSNIIFQNVINL
206	LLOV	Lloviu virus	Cuevavirus		Filoviridae	JF828358	VLREYSWS	RLIGATLPCV	TPYIGSRTED	ILWVT	IVHRYN	DSNIIFQNVINF
207	MBGV	Marburg marburgvirus	Marburgvirus		Filoviridae	Z29337	FLRAYSWL	RLIGATLPCL	APYIGSRTED	LLWVT	IVHRYN	DSNIIFQNTINL
208	RAVV	Ravn virus/Lake Victoria marburgvirus	Marburgvirus		Filoviridae	EU500826	FLRAYSWS	RLIGATLPCL	APYIGSRTED	LLWVT	IVHRYN	DSNIIFQNTINL
209	AMPV-A	Avian metapneumovirus A	Metapneumovirus	Pneumovirinae	Paramyxoviridae	U65312	RIREMSWD	EIVGVSSPSM	APWVGSSTQE	IRWVY	FLHRLA	DINLVFQNAISC
210	AMPV-B	Avian metapneumovirus B	Metapneumovirus	Pneumovirinae	Paramyxoviridae	AB548428	RIRETSWD	EIVGVSSPSM	APWVGSSTQE	IRWVY	FLHRLA	DINLVFQNAISC
211	AMPV-D	Avian metapneumovirus D	Metapneumovirus	Pneumovirinae	Paramyxoviridae	HG934339	KIRESSWD	EIVGVSSPSM	APWVGSSTQE	IRWVY	FLHRLA	DINLVFQNAISC
212	HMPV	Human metapneumovirus	Metapneumovirus	Pneumovirinae	Paramyxoviridae	AF371337	TLRETSWN	EIVGVTSPSI	SPWVGSSTQE	MRWVY	FLHRLS	DINLVFQNAISC
213	AMPV-C	Avian metapneumovirus C	Metapneumovirus	Pneumovirinae	Paramyxoviridae	AY513746	SLRERSWD	EIVGVTSPSI	SPWVGSSTQE	LRWVY	FLHRLS	DINLVFQNAISC
214	HRSV	Human respiratory syncytial virus	Pneumovirus	Pneumovirinae	Paramyxoviridae	M75730	YVRERSWS	NIVGVTSPSI	KPWVGSSTQE	LDWVY	YLHRLT	DIDIVFQNCISF
215	BRSV	Bovine respiratory syncytial virus	Pneumovirus	Pneumovirinae	Paramyxoviridae	AF065167	YVRERSWS	NIVGITSPSI	KPWVGSSTQE	LDWVY	YLHRLT	DIDIVFQNCISF
216	MPV	Murine pneumonia virus	Pneumovirus	Pneumovirinae	Paramyxoviridae	AY729016	IIRDKSWD	DIIGVTSPSP	KPWVGSSTQE	LEWVF	FLHRLS	DINLVFQNAISY
217	CnBV-1	Canary bornavirus 1	Bornavirus		Bornaviridae	KC464471	RLRRLTWG	ELVGVTMPFV	PLYLGSNTAI	YQWYK	LTHRLP	DYTIHFQHVFTY
218	ABBV-1	Aquatic bird bornavirus 1	Bornavirus		Bornaviridae	KF578398	QLRRLTWG	ELVGVSMPFV	PLYLGSNTAI	YQWYK	LTHRLP	DYTIHFQHVFTF
219	BoDV-1	Borna disease virus 1	Bornavirus		Bornaviridae	AJ311522	QLRRLTWG	DLVGVTMPFV	PLYLGSNTAV	HQWYK	LTHRLP	DYTIHFQHVFTY
220	PaBV-1	Parrot bornavirus 1	Bornavirus		Bornaviridae	GU249595	RLRRLTWG	DLIGVTMPFV	PLYLGSNTAI	YQWYK	LTHRLP	DYTIHFQHVLTY
221	LGSV-1	Loveridge's garter snake virus 1			Bornaviridae	KM114265	RLRALTWG	ELVGVTMPFV	PLYLGSNTSV	CEWYK	LTHRLP	DYTIHFQHVFTY
222	NYMV	Nyamanini virus	Nyavirus		Nyamiviridae	FJ554526	ALRAKHWC	DVVGVTMAAP	RPYFGSKTSD	KPWIR	VDHRVG	DWSICYQTLFLA
223	MIDWV	Midway virus	Nyavirus		Nyamiviridae	FJ554525	SLRNRHWS	EIVGVTMAAP	RPYFGSKTSD	KPWIR	VDHRVG	DWSICYQTVFLS
224	SNVV	Sierra Nevada virus			Nyamiviridae	KF530058	RLRARHWF	NPVGITMAAP	RPYFGSKTSE	KPWLG	VDHRVC	DWSICYQTVFVS
225	SCNV	Soybean cyst nematode midway virus			Nyamiviridae	HM849038	QLRQKWWG	DVEGITQAPP	APYLGSQTKE	FEWLR	PTHRLH	NYTVMFQQLFLY

	Abbrev.	Name	Genus	Subfamily	Family	GenBank Acc. No.	A1	A2	B	C	D-like	E
225	VHSV	Viral hemorrhagic septicemia virus	Novirhabdovirus		Rhabdoviridae	Y18263	KLRDLSWG	NIIGVTSPSP	VPYFGTQTKP		ITHKVF	
226	PORV	Paralichthys olivaceus rhabdovirus	Novirhabdovirus		Rhabdoviridae	KC685626	KLRDQSWG	NIIGVTSPSP	VPYFGTQTKP		ITHKVF	
227	SHRV	Snakehead rhabdovirus	Novirhabdovirus		Rhabdoviridae	AF147498	IERDSSWG	NVIGVTSPSP	VPYYGTQTQP		ITHKVF	
228	IHNV	Infectious haematopoietic necrosis virus	Novirhabdovirus		Rhabdoviridae	X89213	ELRDTSWG	NIIGGTSPSP	VPYYGTQTKP		ITHKVF	
229	HIRRV	Hirame rhabdovirus	Novirhabdovirus		Rhabdoviridae	AF104985	GLRDISWG	TIVGVTSPSP	VPYYGTQTKP		ITHKVF	

*: genera proposed

‡: rhabdovirus-like bipartite negative strand RNA viruses
