# Supplementary material for: Signature motifs of GDP polyribonucleotidyltransferase, a non-segmented negative strand RNA viral mRNA capping enzyme, domain in the L protein are required for covalent enzyme–pRNA intermediate formation
Source: Nucleic Acids Res. 2015 Nov 23;44(1):330–41. doi: 10.1093/nar/gkv1286 (PMC4705655; doi:10.1093/nar/gkv1286)
Supplement: SUPPLEMENTARY DATA [file supp_gkv1286_Neubauer-Supplementary-Data-revised-111915.pdf]

Signature motifs of GDP polyribonucleotidyltransferase, a non-segmented negative strand RNA viral mRNA capping enzyme, domain (block V) in the large protein are required for covalent enzyme–pRNA intermediate formation

Julie Neubauer [1, †], Minako Ogino [1, †], Todd J. Green [2], Tomoaki Ogino [1,*]

[1] Department of Molecular Biology and Microbiology, Case Western Reserve University School of Medicine, Cleveland, OH 44106, USA
[2] Department of Microbiology, School of Medicine, University of Alabama at Birmingham, Birmingham, AL 35294, USA.

SUPPLEMENTARY METHODS

Mini-genome assay

The pBS-L plasmid (58) was modified to construct a plasmid expressing a C-terminal FLAG-tagged L protein (pBS-L-Flag). To construct plasmids expressing mutant L proteins, the 5.7-kbp HpaI-HindIII region of the L gene in pBS-L-Flag was replaced with the corresponding HpaI-HindIII fragment derived from the pFastBac L-H plasmids with mutant L genes.

BHK-21 cells (2×10^6 cells) were infected with vTF7-3 (recombinant vaccinia virus expressing T7 RNA polymerase) at a multiplicity of infection of 1, and transfected with pVSV-CAT2 (0.5 µg) along with the supporting plasmids, pBS-N (1 µg), pBS-P (0.5 µg), and pBS-L-Flag (0.3 µg, WT or mutant), using the X-tremeGENE HP DNA Transfection Reagent (Roche Applied Science). The cells were cultured in DMEM supplemented with 5% FBS at 30°C for 2 days in a CO_2 incubator. The cells were lysed and subjected to the CAT ELISA assay (Roche) to analyze the expression levels of CAT. The Flag-tagged L protein in the lysates was detected by Western blotting with an anti-Flag monoclonal antibody (Sigma).

Generation of recombinant VSVs

Recombinant VSVs with the wild-type or mutant *L* gene were generated using the reverse genetics system (58) with some modifications described previously (18).

Amino acid sequence analyses

To find conserved amino acid sequence motifs in block V (putative PRNTase domains) of NNS RNA viral L proteins, block V of the VSV L protein (positions 1078 to 1308) was aligned with those of representative NNS RNA viruses from different genera of the *Mononegavirales* order using the PSI-Coffee program (http://tcoffee.crg.cat/) (50) (Figure S1).

Motifs A–E (Figure S1) were located in block V (putative PRNTase domains) of L proteins of 223 NNS RNA viruses and 2 rhabdovirus-like bipartite negative strand RNA viruses (listed in Table S1) using the CLC sequence viewer program (CLC bio) (data not shown). Local amino acid sequences with motifs A–E in the

225 L proteins (Table S1) were aligned using the WebLogo program (http://weblogo.berkeley.edu) (59) to create sequence logos (Figure S2).

A phylogenetic analysis of amino acid sequences of block V (putative PRNTase domains) of L proteins from selected NNS RNA viruses was performed using the Molecular Evolutionary Genetics Analysis (MEGA) software (version 6.0) (60). A phylogenetic tree was generated using the neighbor-joining algorithm with 1,000 bootstrap repetitions (Figure S4).

SUPPLEMENTARY DISCUSSION

Identification of conserved motifs in putative PRNTase domains

We analyzed amino acid sequences of block V from L proteins of 223 NNS RNA viruses belonging to the different families (*Rhabdoviridae*, *Paramyxoviridae*, *Filoviridae*, *Bornaviridae*, and *Nyamiviridae*) in the order *Mononegavirales* (excluding novirhabdoviruses) and 2 rhabdovirus-like bipartite negative strand RNA viruses (225 viruses listed in Table S1) using the CLC sequence viewer (data not shown) and WebLogo (Figure S2) programs and found that motifs A–E are strikingly conserved in these L proteins.

In motif A [Rx(3)Wx(3-8)ΦxGxζxP], R (R1090 for VSV) is perfectly conserved (225/225 L proteins). W (W1094 for VSV) and G (G1100A for VSV) are also highly conserved, although a minority of the L proteins has Y (PYDV and EMDV, 2/225), R (SalPV; 1/225), or G (SSV, 1/225) and S (OFV; 1/225) at the former and latter positions, respectively. P in motif A is conserved well, but A (LNYV, LYMoV, Sf-RV, NYMV, MIDWV, SNVV, and SCNV; 7/225), T (NIAV and SRIV; 2/225), V (CHOV and SMV; 2/225), or G (FARV; 1/225), is also found at this position. The majority of the L proteins (217/225) have Y (Y1152 for VSV) in motif B [(Y/W)ΦGSxT], while the L proteins of paramyxoviruses belonging to the *Pneumovirinae* subfamily [*e.g.*, HRSV, HMPV] contain W (8/225). Next to this aromatic amino acid residue, a hydrophobic amino acid [*e.g.*, L (143/225), I (30/225), V (25/225), F (17/225)] is found in the majority of known L proteins. G (G1154 for VSV) and T (T1157 for VSV) are perfectly conserved in the 255 L proteins. S (S1155 for VSV) is also well conserved, but a small number of L proteins possess T (3/225), G (2/225), or A (1/225) at this position. Motif C (W, W1188 for VSV) was found to be conserved in L proteins (223/225 L proteins), except for APMV9 and CoRSV. In motif E [ζxxΦx(F/Y)QxxΦ], an aromatic residue [F (140/225), Y (84/225), or H (1/225)] followed by Q (225/225) is strikingly conserved in the 225 L proteins. However, it is interesting to note that some isolates of Newcastle disease virus do not have motif E in their L proteins due to the presence of a frameshift and its compensatory mutation in their genomes (61).

Interestingly, nuclear-replicating NNS RNA viruses (nucleorhabdoviruses, bornaviruses, and nyamiviruses) have the PRNTase domain with motifs A–E, but not the MTase domain, in their L proteins, strongly suggesting that they cap viral mRNAs by their L proteins, but methylate the cap structure using host nuclear MTases. On the other hand, the L proteins of 5 known fish rhabdoviruses (VHSV, PORV, SHRV, IHNV, and HIRRV) in the genus *Novirhabdovirus* possess motifs A and B, but not motif C, D, or E (Table S1). Consistent with this finding, a phylogenetic analysis suggests that their putative PRNTase domains belong to a distinct clade

diverged from an ancestor rhabdoviral PRNTase domain (Figure S4). Although the novirhabdoviral L proteins have been shown to have a motif D-like HK sequence (13,16), it remains unknown whether the HK sequence is a counterpart of motif D (HR motif).

Sequence-specific recognition of mRNA 5′-ends with the VSV PRNTase domain

We have previously found that the VSV and CHPV L proteins forms the L-pRNA intermediate with their mRNA-start sequence (pppAACAG), but not with their leader RNA-start sequence (pppACGAA), in RNA capping (11,17,49). The VSV L protein efficiently caps pppARCNG-initiated RNAs (R and N indicates purine and any nucleotides, respectively), in which the first A and third pyrimidine (C > U) residues are essential (11,17). The same and similar mRNA-start sequences are conserved in many rhabdoviral mRNAs (62-67). Our previous study identified a rhabdovirus-specific R residue located 6 residues upstream of the HR motif as essential for the L-pRNA intermediate formation with the VSV and CHPV L proteins (16,17). Thus, in addition to the conserved amino acid residues, some rhabdovirus-specific amino acid residues are also suggested to be involved in RNA recognition in a sequence dependent manner. R1221 is present on the flexible loop together with catalytic motif D in the cryo-EM structure of the VSV L protein (51), suggesting a possibility that a part of this loop is involved in recognition of a specific base(s) in pppRNA.

Mutations in the HRSV L protein conferring resistance against an anti-capping inhibitor

Liuzzi *et al.* (57) have found that putative small-molecule mRNA capping inhibitors repress *in vitro* mRNA synthesis with HRSV ribonucleoproteins, inducing synthesis of short 5′-triphosphorylated transcripts with less than 50 nt. Although these compounds efficiently inhibited HRSV replication in cultured cells as well as in mice, it was experimentally shown that HRSV becomes resistant to these compounds during passaging in cultured cells in the presence of the compounds. Interestingly, some HRSV mutants resistant to these compounds were found to possess amino acid substitutions (E1269D and I1381S) very close to motif B (1262-WVGSST-1267) and within motif E (1380-DIDIVFQNCI-1389), respectively, of the HRSV L protein. These observations suggest essential roles of these motifs in pre-mRNA capping with other viral L proteins.

SUPPLEMENTARY FIGURES

```
VSV      1078 MWTCSATHADTLRYKSWG-----RTVIGTTVPHPLEMLGPQH-RKET-PCAPCNTS-----------------
SVCV     1066 IWECSSTQADLLREKSWG-----RSVIGMTVPHPLEMFGKGH-QKEL-ECIPCQTS-----------------
PRV      1096 IWSCSSTRADELRQESWG-----TKVIGTTVPHPIEMINCSH-TGGT-C---QQDE-----------------
BEFV     1105 IWDCSASLADSLRKRSWG-----KEVLGTTVPHPAEMFKGYR-GGED-SCSFCRGNGS---------------
TIBV     1095 IWTCSAAHADKLRKQSWN-----EDIIGMTVPHPIEMHKIGY-VVNG-ECLHCYENKL---------------
TUPV     1074 PWNCSASHADRLRRLSWG-----QPVIGATIPHPFEMLGKVC-YLFR-GSGCECP--D---------------
DMeISV   1081 MWPCSSTQADYLRRVSWQ-----QVVYGATIPHPAELFGLPL-RAAP-TCPNCTTTFP---------------
RABV     1090 VWPCSSERADLLREISWG-----RKVVGTTVPHPSEMLGLLP-KSSI-SCTCGATG-G---------------
SYNV     1139 IGSCPTRDSKMLRNWTWG-----KNIIGVTTPHPLGYLKRER-HSESSSCDN---------------------
NCMV     1055 VSLCPSEQIRHLRKKGWK-----KDVVGISTPYPHHFLGGED-ETDR-P------------------------
MuV      1139 VDTCSIDIARSLRKLSWATLLNGRPIEGLETPDPIELVHGCLIIGSD-ECEHCSSGD----------------
NDV      1109 SNMCSLTLADYARNRSWSPLTGGRKILGVSNPDTIEPVEGEILSVSG-GCKKCDSGD----------------
MeV      1129 KESCSVQLARALRSHMWARLARGRPIYGLEVPDVLESMRGHLIRRHE-TCVICECGS----------------
NiV      1188 SNTCSVDLARALRSHMWRELALGRVIYGLEVPDALEAMVGRYITGSL-ECQICEQGN----------------
FDLV     1124 SLVCSVTLAKIMRSRMWVKVAKGRRIEGLEVPDPLECLRGELIVGSG-SCTICERGM----------------
SeV      1129 EYMCSVELAVGLRQKMWIHLTYGRPIHGLETPDPLELLRGIFIEGSE-VCKLCRSEGA---------------
ASPV     1132 STYCSVDLAKIIRTKLWCSLSSGRHIAGLETPDPLEVINGQIIKGHE-MCSLCESEGE---------------
HRSV     1195 ENLSITELSKYVRERSWSL----SNIVGVTSPSIMYTMDIKY-TTST-I------------------------
HMPV     1120 GRLICCQISRTLRETSWNN----MEIVGVTSPSITTCMDVIY-ATSS-H------------------------
ZEBOV    1102 QITCTVDLAQILREYSWAHILEGRPLIGATLPCMIEQFKVFWLKPYE-QCPQCSNAKQP--------------
MBGV     1105 KFTCTVDIANFLRAYSWLDVLKGKRLIGATLPCLLEQFKVKWINLSE-DLREQFNMSSESESTINLLPYDCKE
LLOV     1099 QLTCTLDIANVLREYSWSHVLQGRRLIGATLPCVPEQFELTWLTGDK-ACKHCQSKLRD--------------
BDV       990 LEGCTYLAAKQLRRLTWG-----RDLVGVTMPFVAEQFHPHS-SVGAKAELY---------------------
NYMV     1035 GPWCSALAAQALRAKHWCE----YDVVGVTMAAPQEQFLLAP-YSELPVEAY---------------------

cons                      *   *        : *   .
                                   A


VSV      1127 ---------GFNYVSVHC-------PDGIHD--VFS-SRGPLPAYLGSKTSESTSILQPWERESKVPLIKRAT
SVCV     1115 ---------GLTYISSY--------CPRGIN-NWYS-TVGSLAAYLGSKTSETTSILQPWEKDSKIPIIKRAT
PRV      1136 ---------VLDYINVSI-------VQDLRN--CLN-SKGKVPAYLGSKTSETTSILQPWEKETKIPVIKRAA
BEFV     1156 ---------NNNYLTVLM-------PRGIPM--KCH-YRGPYYPYLGSNTKESTSILQPWEKETKVPVLKRAC
TIBV     1146 ---------SQCYISILT-------PRGIPNTDYYC-EGGPYKPYLGSSTNEGTSVLQPWEKETKIPIIRRAA
TUPV     1123 ---------SSNYTTTFV-------NWDAES--VMS-RKGPFLPYLGSKTSESTSLINPWERETIIPLIKRAA
DMeISV   1132 ---------MNLYISVLI-------PLGFKG--LKD-TRGTCVAYLGSSTTESTGIVNPWEKEAVVPVIKRAA
RABV     1140 ---------GNPRVSVSV-------LPSFDQ--SFF-CTGPLKGYLGSSTSMSTQLFHAWEKVTNVHVVKRAL
SYNV     1185 -----------NYIRVLT-------KRIGNS--WEL-RRGQFRPYFGSYTEEKFKMTTLASAYGDESILKRAI
NCMV     1097 ----------DSYVEVVVNDV---VLSHPDR--LIL-TTGSSLPYLGSVTKEKLHSTSARAAYGTEPLITRPI
MuV      1195 ----------DKFTWFFLPKGI-----RLDN--DPASNPPIRVPYIGSKTDERRVASMAYIKGA-SVSLKSAL
NDV      1165 ----------EQFTWFHLPSNI-----QLTD--DTSKNPPMRVPYLGSKTQERRAASLAKIAHM-SPHVKAAL
MeV      1185 ----------VNYGWFFVPSGC-----QLDD--IDKETSSLRVPYIGSTTDERTDMKLAFVRAP-SRSLRSAV
NiV      1244 ----------TMYGWFFVPRDS-----QLDQ--VDREHSSIRVPYVGSSTDERSDIKLGNVKRP-TKALRSAI
FDLV     1180 ----------DQYSWFYIPPGM-----DLEG--QHRENGSLRVPYFGSSTSERSEASVNTTRGL-SRAAKAAI
SeV      1186 ---------DPIYTWFYLPDNI-----DLDT--LTNGCPAIRIPYFGSATDERSEAQLGYVRNL-SKPAKAAI
ASPV     1189 ---------DKRYSWFFIPKDI-----NLED--LSKATESIRVPYFGSTTEERSEIRLGTPRP--VKAGEGCI
HRSV     1238 ----------SSG--IIIEK-----YNVNSL--TRG-ERGPTKPWVGSSTQEKKTMPVYNRQVL-TKKQRDQI
HMPV     1163 ----------LKG--IIIEK-----FSTDRT--TRG-QRGPKSPWVGSSTQEKKLVPVYNRQIL-SKQQREQL
ZEBOV    1160 --------GGKPFVSVAVKKHIVSAWPNASR--ISW-TIGDGIPYIGSRTEDKIGQPAIKPKCP-SAALREAI
MBGV     1177 LRLGRSNDTELNYVSCALDRKVVQKHPSVNR--LAW-TIGNRAPYIGSRTEDKIGYPPLRVNCP-SAALKEAI
LLOV     1157 --------KKKPYVSAALVDKIISQRPSYHR--LSW-TIGTLTPYIGSRTEDKIGQSAFKPRCP-SAALRETI
BDV      1036 ---------LDAIIYCPQ-------ETLRSH--HLT-TRGDQPLYLGSNTAVKVQRGEITGLT-KSRAANLVK
NYMV     1082 ---------PDTLL-ACV-------ESLEPN--AYL-TRGKFRPYFGSKTSDKVKRGTLQIVD-ADKQLVALR

cons                                                      :.** *
                                                             B
```

(continued)

```
VSV       1181 RLRDAISWFVEPD---SKLAMTILSNIHSLTGEEWTK--RQHGFKRTGSALHRFSTSRMSHGGFASQSTAALT
SVCV      1169 KLRDSISWFVPPD---SKLAKSIQQNLKALTGEDWEE--DIQGFKRTGSALHRFTTSRVSNGGFSAQSPAKLT
PRV       1190 KLRAAITWFVEPE---SDLSNSILNNISSLTGEDWSG--SIQGFKRTGSALHRFTSARVSAGGFAAQSPARLT
BEFV      1210 DLRKSINWFVTPD---SLLAKSIFNNLKALTGEDWED--QIKGYKRTGSSLHRFGCSRVSSGGFSASSPSCFT
TIBV      1202 RLRDVISWFVGED---SNLSESIINNLEALTGENWGN--YMRGFKRTGSALHRFRCARVSNGGFSACSPTKSS
TUPV      1177 KLRNAINWFVRSD---SLLARSILNNLRALTGEDPGQ--GNPGFFRTGSALHRFACSRQSSGGFSALSPAYLS
DMeISV    1186 SLRNGIGWFIEPG---SNLAQSILNNLQSLTGESWSQ--NSGGVRRTGSALHRFSCSRQSSGGYTAQNPSKLT
RABV      1194 SLKESINWFITRD---SNLAQTLIRNIVSLTGPDFPLE-EAPVFKRTGSALHRFKSARYSEGGYSSVCPNLLS
SYNV      1237 KIQKLLGWRYHQG---SSLYNLIQKILTCVTDADPNKFL-PLPDEITGDVEHRYHDMATKHGGIPSNLIHLYT
NCMV      1154 KLLRAIGWFIDEE---SNWAESIRNLLKAVTDLDPGKVI-SIPEHVKGSMMHRYLDMALAHGSLWMPSFGPAS
MuV       1250 RLAGVYIWAFGDT---EESWQDAYELASTRVNLTLEQLQSLTPLPTSANLVHRLDDGTTQLKFTPASSYAFSS
NDV       1220 RASSVLIWAYGDN---EVNWTAALKIARSRCNISSEYLRLLSPLPTAGNLQHRLDDGITQMTFTPASLYRVSP
MeV       1240 RIATVYSWAYGDD---DSSWNEAWLLARQRANVSLEELRVITPISTSTNLAHRLRDRSTQVKYSGTSLVRVAR
NiV       1299 RIATVYTWAYGDN---EECWYEAWYLASQRVNIDLDVLKAITPVSTSNNLSHRLRDKSTQFKFAGSVLNRVSR
FDLV      1235 RIASVYTWAYGES---QLSWYEAYALASQRANLTLEQLKVITPKSTSTSLSHRLNDSSTQMKFASTNLQRVSR
SeV       1242 RIAMVYTWAYGTD---EISWMEAALIAQTRANLSLENLKLLTPVSTSTNLSHRLKDTATQMKFSSATLVRASR
ASPV      1244 RIAMVYTWAFGDD---EISWEEASTIAKTRANFTTEELKLLTPASTSTNLSHRLRDSSTQMKFAGTSLLRVSR
HRSV      1290 DLLAKLDWVYASI---DNKDEFMEELSIGTLGLTYEKAKKLFPQYLSVNYLHRLTVSSRPCEFPASIPAYRTT
HMPV      1215 EAIGKMRWVYKGT---PGLRRLLNKICLGSLGISYKCVKPLLPRFMSVNFLHRLSVSSRPMEFPASVPAYRTT
ZEBOV     1221 ELASRLTWVTQGS---SNSDLLIKPFLEARVNLSVQEILQMTPSHYSGNIVHRYNDQYSPHSFMANRMSNSAT
MBGV      1246 EMVSRLLWVTQGT---ADREKLLIPLLNSRVNLDYQTVLNFLPTHYSGNIVHRYNDQYGQHSFMANRMSNTST
LLOV      1218 ELASRILWVTQGS---SQAEDIIRPFCEARINLPVQELFKLLPSHYSGNIVHRYNDQYGPRSFMANRMSNTAT
BDV       1089 DTLVLHQWYKVRKVTDPHLNTLMARFLL---EK--GYTSDARPSIQGGTLTHRLPSRGDSRQGLTGYVNILST
NYMV      1134 KILTLKPWIRSQD--CPNLESLLNLLIEEKTSIPLSDLEILKDLRISGKVDHRVGNPTSPKGSMANTLLGFSS

cons                  *                                            **
                      C                                            D


VSV       1249 RLMATTDTMR---DLG-----DQNFDFLFQATLLYAQITTTVA--R--DGWITSCTDHYHIACKS-CLRPIEE
SVCV      1237 RIMTTTDTMR---DLG-----DQNYDFMFQAGILYSQMTTGEL--R--ENSTNSTATHYHITCKS-CLREIQE
PRV       1258 RMMATTDTFR---EIG-----SDNYDFMFQSLLLYSQMTAGEL--Y---GDNPTTVYHFHLSCKK-CLRKIEE
BEFV      1278 WCIATTDTMC---GLG-----EVNYDFMFQSTLVWCQMSSIIR--E--RGNLHSKIHHYHIKCNK-CLREIQE
TIBV      1270 WMIVTTDTMT---GLD-----ESNYDFMFQASIIFSQVTVGSI--Q----RSTSQIYHMHLNCQE-CLREIKE
TUPV      1245 RFLTTTDTLQ---GIG-----DRNYDFMFQSLILYSQSSLCV---Q--INRNVQGIVHHHISCNE-CLREITE
DMeISV    1254 RMIATTNYLA---DLG-----DENYDFMYQSCLLNALISVGEI--H--PVDGSQGYYHQHVNCAS-CLRPIKE
RABV      1263 HISVSTDTMS---DLT---QDGKNYDFMFQPLMLYAQTWTSELVQR--DTRLRDSTFHWHLQCNR-CVRPIDD
SYNV      1306 HASCNTSTFI---NHS---KGAANESLHFQAAIIWTCMQSICRT-S--ASSSVSDISHYHEACNQ-CIVKLED
NCMV      1223 HLSMSTNTLL---EYA---KGSKNVTLQFQAMLGL--IQFCTINRL--LSSEPRKIVRVYRTCPH-CIKPVDE
MuV       1320 FVHISNDCQV---LEID--DQVTDSNLIYQQVMITGLALIETWNNPPINFSVYETTLHLHTGSSC-CIRPVES
NDV       1290 YIHISNDSQR---LFTE--EGIKEGNVVYQQIMLLGLSLIESLFPMTTTKTYDEITLHLHSKFSC-CIREAPV
MeV       1310 YTTISNDNLS---FVIS--DKKVDTNFIYQQGMLLGLGVLETLFRLEKDTGSSNTVLHLHVETDC-CVIPMID
NiV       1369 YVNISNDNLD---FRIE--GEKVDTNLIYQQAMLLGLSVLEGKFRLRLETDDYNGIYHLHVKDNC-CVKEVAD
FDLV      1305 FVTISNDHIK---LKGN--GDAGDTNLIYQQVMLVGLSIIELTHRRQENTGEDSKVLHLHIREDC-CVEFTQD
SeV       1312 FITISNDNMA---LKEA--GESKDTNLVYQQIMLTGLSLFEFNMRYKKGSLGKPLILHLHLNNGC-CIMESPQ
ASPV      1314 YVTISNDQMV---MKEA--GESKDTNLVYQQVMLIGLSFLEYLSRHESTTGDENTLLHLHLNPGC-CIVTNED
HRSV      1360 NYHFDTSPINRILTEK---YGDEDIDIVFQNCISFGLSLMSVVEQF--TNVCPNRIILIPKLNEIHLMKPP-I
HMPV      1285 NYHFDTSPINQALSER---FGNEDINLVFQNAISCGISIMSVVEQL--TGRSPKQLVLIPQLEEIDIMPPP-V
ZEBOV     1291 RLIVSTNTLG---EFSGGGQSARDSNIIFQNVINYAVALFDIKFRNTEATDIQYNRAHLHL-TKC-CTREVPA
MBGV      1316 RAIISTNTLG---KYAGGGQAAVDSNIIFQNTINLGVAVLDIALSLAKLSSASNVTFRLML-NKC-CTRHVPS
LLOV      1288 RIIVSTNTLG---PYSGGGQAARDSNIIFQNVINFAVAVLDIQNSFLDQPMSEFKHIHLHI-KDC-CTREVPS
BDV       1157 WLRFSSDYLH---SFS---KSSDDYTIHFQHVFTYGCLYADSVI-R--SGGVISTPYLLSASCKT-CFEKIDS
NYMV      1205 HIFITTDTAT---NQT---RGGQDWSICYQTLFLAAISRLELLN-R--FRVPIQGKWGLWTDCRG-CTRPVND

cons                ..                :  . :*  :
                                            E
```

Figure S1. Amino sequence alignment of putative PRNTase domains of NNS RNA viral L proteins.

The amino acid sequences of block V (PRNTase domain) of representative NNS RNA viral L proteins (see Figure 1) were aligned. Virus names are abbreviated as shown in Table S1. Conserved amino acid residues in motifs A–E are highlighted.

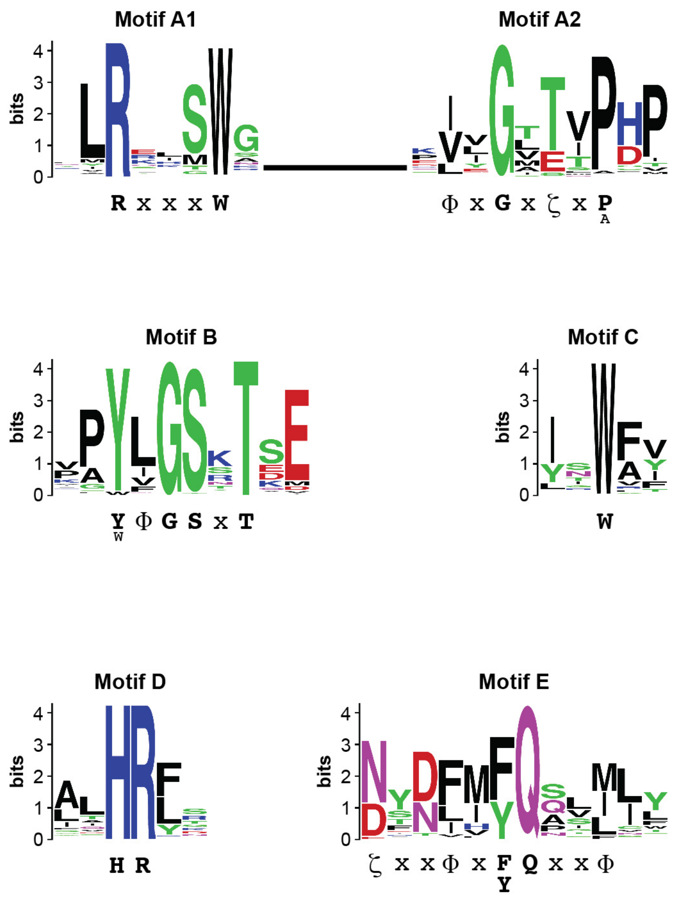

Figure S2. WebLogo sequence alignment of conserved amino acid sequences in putative PRNTase domains.

Local amino acid sequences (including the motifs A–E) in PRNTase domains of 225 L proteins (listed in Table S1) were analyzed by the WebLogo program. Two regions (referred to as A1 and A2) in motif A were separately analyzed. Highly conserved amino acids at indicated positions are shown in bold letters under the logos, whereas minor amino acids are in regular letters. x, Φ, and ζ indicate any, hydrophobic, and hydrophilic amino acids, respectively.

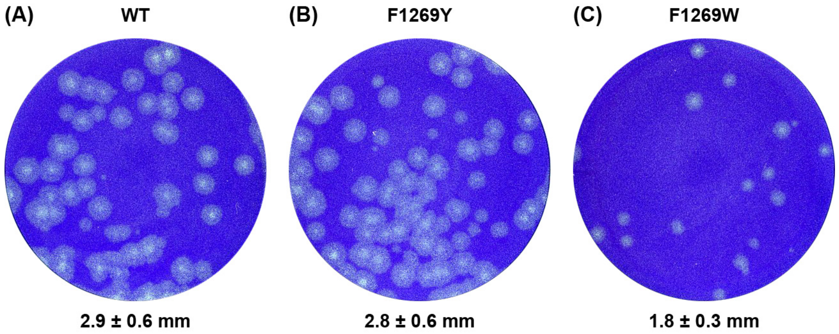

Figure S3. Plaque phenotypes of recombinant VSVs with the F1269F and F1269W mutations.

Recombinant VSVs with the F1269Y (B) and F1269W (C) mutations were generated using a reverse genetics system. Their plaque phenotypes were compared with that of the WT recombinant virus (A). Twenty plaques were randomly chosen from wells of the same plate to measure their sizes (the mean ± standard deviation).

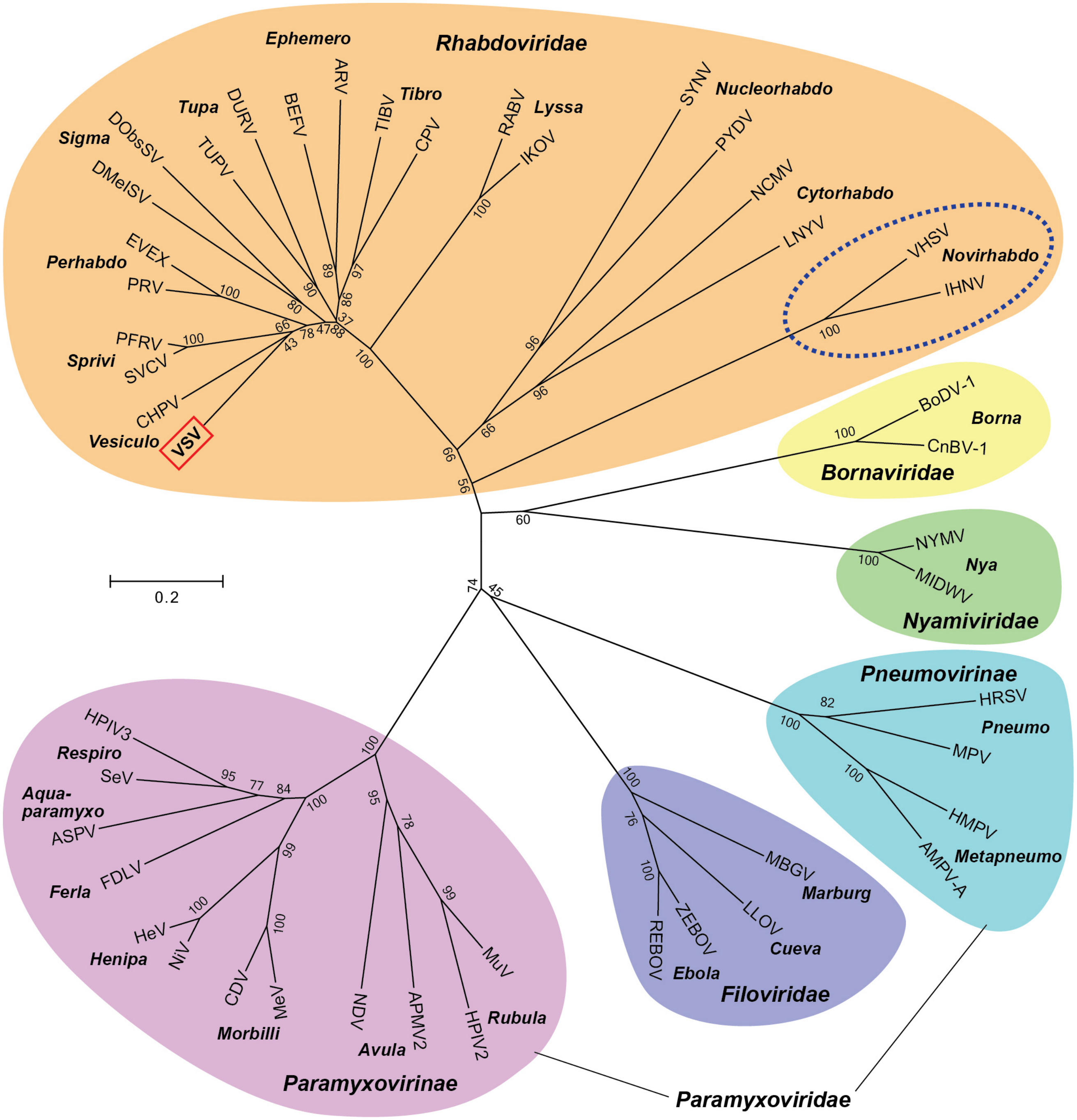

Figure S4. Phylogenetic analysis of putative PRNTase domains in NNS RNA viral L proteins.

Block V (putative PRNTase domain) of L proteins from VSV (highlighted by a red square) and selected NNS RNA viruses was analyzed using the MEGA6.0 software to generate a phylogenetic tree. Virus names are abbreviated as shown in Table S1. Colored branches represent different families and subfamilies. Genus names are placed close to the virus names. The numbers at the nodes indicate bootstrap values. The scale bar shows the branch length corresponding to 0.2 amino acid substitutions per site. Block V of known L proteins of NNS RNA viruses belonging to different families contains conserved motifs A–E, except that L proteins of known novirhabdoviruses (indicated by a dashed blue oval) possess motifs A, B, and a motif D-like sequence (see Table S1).

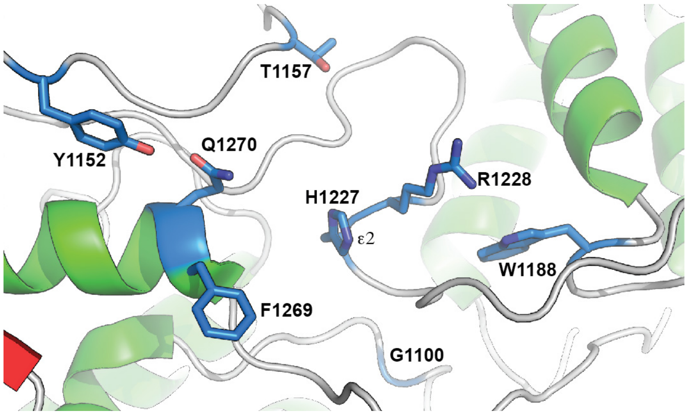

Figure S5. Localization of the amino acid residues required for the L-pRNA intermediate formation in the putative PRNTase domain.

The structure of the PRNTase active site shown in Figure 8A is viewed from a different angle.

SUPPLEMENTARY REFERENCES

58. Lawson, N.D., Stillman, E.A., Whitt, M.A. and Rose, J.K. (1995) Recombinant vesicular stomatitis viruses from DNA. *Proc. Natl. Acad. Sci. U. S. A.*, **92**, 4477-4481.
59. Crooks, G.E., Hon, G., Chandonia, J.M. and Brenner, S.E. (2004) WebLogo: a sequence logo generator. *Genome Res.*, **14**, 1188-1190.
60. Tamura, K., Stecher, G., Peterson, D., Filipski, A. and Kumar, S. (2013) MEGA6: Molecular Evolutionary Genetics Analysis version 6.0. *Mol. Biol. Evol.*, **30**, 2725-2729.
61. Kusumaningtyas, E., Tan, W.S., Zamrod, Z., Eshaghi, M. and Yusoff, K. (2004) Existence of two forms of L protein of Newcastle disease virus isolates due to a compensatory mutation in Domain V. *Arch. Virol.*, **149**, 1859-1865.
62. Bourhy, H., Kissi, B. and Tordo, N. (1993) Molecular diversity of the Lyssavirus genus. *Virology.*, **194**, 70-81.
63. Hoffmann, B., Schutze, H. and Mettenleiter, T.C. (2002) Determination of the complete genomic sequence and analysis of the gene products of the virus of Spring Viremia of Carp, a fish rhabdovirus. *Virus Res.*, **84**, 89-100.
64. Marriott, A.C. (2005) Complete genome sequences of Chandipura and Isfahan vesiculoviruses. *Arch. Virol.*, **150**, 671-680.
65. McWilliam, S.M., Kongsuwan, K., Cowley, J.A., Byrne, K.A. and Walker, P.J. (1997) Genome organization and transcription strategy in the complex GNS-L intergenic region of bovine ephemeral fever rhabdovirus. *J. Gen. Virol.*, **78**, 1309-1317.
66. Galinier, R., van Beurden, S., Amilhat, E., Castric, J., Schoehn, G., Verneau, O., Fazio, G., Allienne, J.F., Engelsma, M., Sasal, P. *et al.* (2012) Complete genomic sequence and taxonomic position of eel virus European X (EVEX), a rhabdovirus of European eel. *Virus Res.*, **166**, 1-12.
67. Walker, P.J., Firth, C., Widen, S.G., Blasdell, K.R., Guzman, H., Wood, T.G., Paradkar, P.N., Holmes, E.C., Tesh, R.B. and Vasilakis, N. (2015) Evolution of genome size and complexity in the rhabdoviridae. *PLoS Pathog.*, **11**, e1004664.
